# Benefits beyond health in the willingness to pay for a quality-adjusted life-year

**DOI:** 10.1007/s10198-024-01726-7

**Published:** 2024-10-07

**Authors:** Linda M. de Vries, Werner B. F. Brouwer, Pieter H. M. van Baal

**Affiliations:** Erasmus School of Health Policy and Management, Department of Health Economics, Erasmus University Rotterdam, P.O. Box 1738, 3000 DR Rotterdam, The Netherlands

**Keywords:** Willingness to pay, QALY, Utility of consumption, Societal perspective, Cost-effectiveness analysis, I310

## Abstract

Adopting a societal perspective in cost-effectiveness analysis (CEA) requires including all societal costs and benefits even if they fall outside of the realm of health and healthcare. While some benefits are not explicitly included, they might be implicitly included when people value quality-adjusted life-years (QALYs) in monetary terms. An example is utility of consumption (UoC) which has played a crucial role in discussions regarding the welfare economic underpinnings of CEA. This study investigates whether people consider elements beyond health when valuing QALYs monetarily and the influence of inclusion on this value. A Willingness to Pay (WTP) experiment was administered among the general public in which people were asked to assign monetary values to QALYs. Our results show that (stated) UoC increases with quality of life but that instructing people to consider UoC does not impact their monetary valuation of the QALY. Furthermore, many respondents consider elements beyond health when valuing QALYs but the impact on the monetary value of a QALY is limited. These findings suggest that these elements are currently not (adequately) captured in CEA. Findings also illustrate that it is difficult to isolate health from non-health benefits and to consistently capture these in CEA. With that, reconciling CEA with welfare economics remains challenging.

**Supplementary Information:**

The online version contains supplementary material available at 10.1007/s10198-024-01726-7.

## Introduction

Cost-effectiveness analyses (CEAs) are increasingly used to aid decision makers in healthcare in the allocation of scarce resources [1]. CEAs provide information on the costs and benefits of an intervention compared to a relevant reference case and are typically summarized in the incremental cost-effectiveness ratio (ICER), the ratio of additional monetary costs to additional benefits of one intervention compared to another, which can subsequently be compared to a threshold to determine whether the intervention yields sufficient value for money. This paper dives into the discussion on the extent to which current CEAs, including the threshold, capture benefits beyond health that are related to changes in health. This, to provide researchers and decision makers with more information on what benefits CEAs currently capture and to provide directions on how to improve (decision making based on) CEAs such that all relevant information is adequately measured and valued.

The most frequently applied form of CEA is cost-utility analysis in which benefits are quantified in quality-adjusted life-years (QALYs), life-years corrected for health-related quality of life [2]. CEAs can be performed from different perspectives, of which the healthcare perspective, where only costs falling on the healthcare budget and health benefits are considered, and the broader societal perspective, where all relevant costs and benefits for society are considered, are most frequently applied [3]. This broader perspective takes into account that investments in health may have implications beyond health and that its value lies not only in the intrinsic value of health (obtaining value from being healthier) but also in the instrumental value of health (the way in which improved health enables or disables one to obtain value in other areas of life, such as work and sports). The standard decision rule related to CEAs states that an intervention should only be adopted if the ICER is below a threshold representing a relevant monetary value of health [4]. The nature of what the threshold for ICERs represents differs between the perspectives: it represents the health losses due to displaced healthcare spending when adopting a healthcare perspective and the consumption value of health when adopting a societal perspective [4, 5].

It may be considered most appropriate to take a broader societal perspective in CEAs when the aim of the decision maker is to optimize social welfare [6]. Formalized, the relevant decision rule may then be written as:

1 $$v_Q \Delta Q - \Delta c_t > 0.$$

In which ${v}_{q}$ denotes the consumption value of the (health or broader) outcome Q, now often expressed as QALYs, and ${c}_{t}$ denotes total costs. This equation highlights that the total value of the gains (value times quantity) minus total societal costs required to produce that value should be larger than zero. Rewriting Eq. (1) gives the ICER expression:

1′ $$\frac{\Delta c_t }{{\Delta Q}} < v_Q .$$

Equation (1′) simply states that the costs per produced unit of outcome should not exceed the value per unit.

Adopting such a broad societal perspective suggests including all relevant costs and benefits in the analysis even if they fall outside of the realm of health and healthcare (budgets) [7]. Nonetheless, there has been an extensive debate on the extent to which different cost categories should be included in cost effectiveness analyses [8–10]. An important line of reasoning in this debate has been that costs should only be included when the related benefits are also captured in the evaluation—since every transaction has a cost and benefit side and what matters from an economic welfare perspective is the net effect [8]. As an example, it would be inconsistent to include additional costs of food resulting from a medical intervention when the utility gains brought by this consumption would not be accounted for. In the same way, it would be inconsistent to include the production gains from working more hours while not considering the utility loss from giving up leisure. (Note that while consistency is important, so is the normative viewpoint that *all* costs and benefits need to be included in economic evaluations—suggesting that the optimal consistency is that in which all benefits and costs are indeed captured—which may therefore also require broader benefit measures if the QALY does not capture everything of value).

In many ways, what benefits are captured in CEAs is an empirical question and although the QALY has been developed specifically as an outcome measure that captures the benefits of improvements in *health*, it may implicitly also capture other (or broader) benefits. In this context, several researchers investigated what people considered when asked to evaluate changes in health in the process of assigning QALY weights to descriptive health states. As an example, in one of these studies it was found that 60% of the respondents who did not receive explicit instructions to do so, spontaneously considered utility of consumption and leisure in time-trade-off exercises [11]. Explicit instructions to consider the elements did not substantially influence outcomes. However, spontaneous consideration without explicit instruction did significantly affect valuations. Thus far, research into the benefits beyond health captured in CEAs focused on the measurement and weighting of QALYs in relation to potentially included cost elements. Surprisingly, comparable attention has not been given to the inclusion of these benefits when investigating the monetary value of the QALY. However, as shown in Eq. (1), it is the combination of the QALY and the monetary value of the QALY that comprises the benefit side in a traditional cost–benefit framework on which current CEA are predominantly based. After all, it is the threshold that represents the value by which to judge whether an intervention is deemed cost-effective (i.e., improves welfare) [12, 13]. The consumption value, or monetary value of QALY gains, is often approximated using Willingness to Pay (WTP) exercises, in which people are asked how much money they are willing to pay for (hypothetical) gains in length or quality of life [14]. This information can be used to derive a monetary value of a QALY. Several of such studies have been performed for the Netherlands (and other countries) with different approaches and focus points (e.g., [12, 13]). Note that available estimates differ substantially, also in relation to respondent characteristics and methods applied [14, 15].

Important for the current context is that in valuing gains in health through WTP exercises respondents may include broader aspects than health alone. To our knowledge, this has not yet been investigated. Nonetheless, to obtain more insight into what benefits beyond health are currently captured in the full decision framework of CEAs, it is important to consider the framework as a whole and thus to also pay attention to those elements considered in the monetary value of the QALY, in addition to what is considered in the measurement and weighting of QALY gains (i.e., in health state valuations and QALY tariffs). In this, not only should attention be paid to which elements are considered when quantifying and valuing outcomes in terms of Q and ${v}_{Q}$ in Eq. (1), but also how they might interact. Although in the current CEA framework it is typically assumed that changes in health measured in QALYs are independent from changes beyond health such as increases in leisure and/or income, people may still consider such elements and their expectations for, and valuation of these elements might also depend on the health state considered. Here, there may also be variation in different types of QALY gains. For instance, it may be that inclusion of and expectations about elements vary depending on whether the QALY gain results from a change in quality or length of life. This all relates to health-state dependency, which can be seen as the change in marginal utility of consumption with health status, an important theme in the (health-)economic literature [16–18]. These aspects do not only have implications for the inclusion of costs following from the general rule underlying the consistency principle that cost should be included when benefits are, but might also have more fundamental implications as these aspects might violate welfare economic underpinnings of CEA [19]. For instance, a crucial assumption that is needed for CEA to be consistent with cost–benefit analysis is that the utility of consumption is independent from health [19].

The aim of this study is to investigate the extent to which people consider elements beyond health when valuing QALYs in monetary terms using a WTP questionnaire. The setup in our study is comparable to earlier research into elements considered in the process of assigning QALY weights to descriptive health states (e.g., [11, 20]). We asked respondents after answering WTP questions whether they had considered utility from consumption, leisure time, spending patterns, and productivity during the exercises. We also investigated what they expected from these elements in relation to changing health, both in terms of quality of life and length of life, also obtaining a direct measurement of expected utility of consumption in different health states (i.e., health state dependency). To investigate the impact of explicit instructions to include elements beyond health, we also asked part of the respondents to consider the utility from consumption when assigning monetary values to health states. We focus on the utility of consumption given the role thereof in the welfare economic underpinning of CEA as well as in the debate regarding the inclusion of costs of non-medical consumption in CEA [8, 9, 19], aiming to provide more insight into the extent to which the utility of consumption is independent from health and whether it might be implicitly included when people value QALYs in WTP exercises.

The structure of this paper is as follows. We describe the methods applied in the current study in the following methods section, followed by the results and the discussion and conclusion.

## Methods

### Sample and data collection

We designed a contingent valuation study in which we elicited peoples WTP for a QALY from an individual perspective. We additionally obtained information on the extent to which they considered benefits beyond health during these tasks and information on the utility of consumption related to different health states. A total of 1159 respondents were sampled to be representative of the Dutch general public by age (18–75 years), sex, and education level. The survey was administered online by a professional research agency (Dynata) in November and December 2020.^1^ On completion of the questionnaire, people received points to exchange for a discount, gift card or donation to charity.^2^ The dataset generated by the survey research and analyzed during the current study is available in the EUR Data Repository at 10.25397/eur.24926226.

### Survey design

**Fig. 1:**
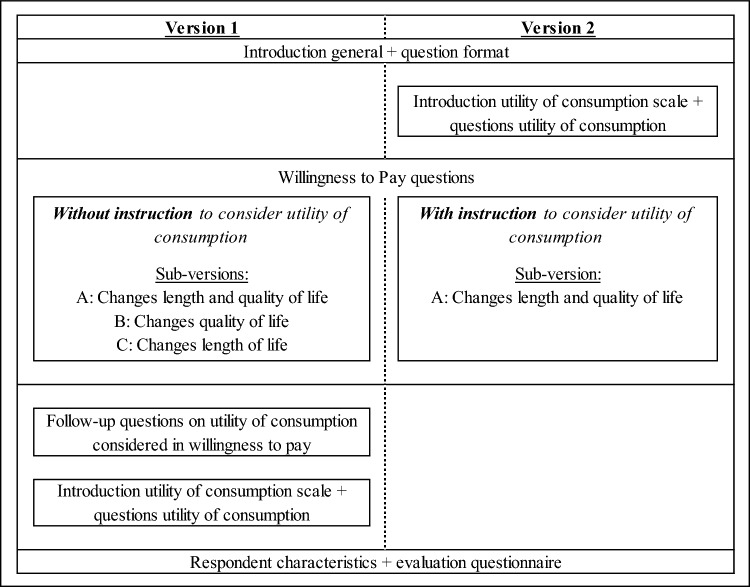
Summary survey design

Two versions of the questionnaire were developed. One version without explicit instructions (Version 1) and one version with explicit instructions (Version 2) to consider utility of consumption during the WTP tasks. A summary of the survey design is shown in Fig. 1. For both versions, we provided a brief explanation of the purpose and content of the research and asked for informed consent at the start of the questionnaire. We then introduced the visual analogue scale (VAS), a scale ranging from 0 (dead) to 100 (completely healthy), which we used to describe health states in the WTP tasks [21]. We asked people to use the VAS to rate their own health to familiarize them with the scale. We also introduced respondents to the way in which health changes and the related hypothetical treatments would be presented in the WTP tasks. In Version 2, we explained the concept of utility of consumption (UoC) and introduced the UoC scale at this point as this scale would be used in the instruction to consider UoC during the WTP tasks. UoC was explained as “the satisfaction someone experiences when purchasing and consuming goods and services”. The UoC scale was constructed for the purpose of this research. To avoid complexities and burden on respondents, it was designed to be highly comparable to the VAS scale, with a range from 0 (no utility) to 100 (highest utility achievable). The introduction of the term utility of consumption and an image of the UoC scale can be found in “Introduction utility of consumption and utility of consumption scale” in the appendix. We constructed this scale to obtain information on how UoC is expected to change with health and to help respondents to consider UoC in their valuations in Version 2. For this, we asked people to rate their own UoC and what they expected their UoC would be in the health states used as health states before and after treatment in the WTP tasks. We also used peoples own UoC and own QoL to obtain information on how UoC is related to QoL and other factors, next to its purpose of familiarizing respondents with the scales. We asked people to consider their current income here and during the WTP tasks.

In the WTP tasks that followed upon the introduction, people were asked to state their WTP for a 0.25 QALY gain as the result of a painless medicine without side effects, to be paid in twelve monthly installments. The QALY gain was both described in words and shown on the VAS. For Version 2, we additionally showed the UoC scale with the UoC values corresponding with the QALY scores based on the earlier valuation by the respondent. An example of the WTP tasks can be found in “WTP scenario version 1” in the appendix for Version 1 and in “WTP scenario Version 2” in the appendix for Version 2. For the WTP answers, we applied a two-step elicitation method, using a payment scale followed by a bounded direct open-ended follow-up question, to combine the ease of the payment scale with the precision off with the precision of an open-ended format. First, we asked what people would certainly be willing to pay, using a payment scale ranging from 0 to > 2500 (if > 2500 was chosen, additional options were presented). Then, we asked what they would certainly not be willing to pay using the same payment scale. Finally, we asked in an open-ended question for the amount that most accurately described their WTP within the range of numbers from the previous questions. This scale was adopted from and tested in earlier research [12]. Respondents who chose €0 as their WTP were asked why, also to detect protest answers.

Different subversions of the questionnaire were constructed based on the scenarios in which the 0.25 QALY gain occurred, to which respondents were randomly assigned. Health gains were presented on different points on the QALY scale as previous research has shown that the health before start of the treatment can affect the WTP [22]. In the same way, both improvements in QoL and length of life are valued [14]. In Version 1A and 2A, people were asked to value a QoL gain from 0.25 to 0.50 for 1 year, a QoL gain from 0.75 to 1 for 1 year, an extension of life of 1 year in QoL 0.25, and an extension of life of 4 months in QoL 0.75. In Version 1B, people were asked to value a QoL gain from 0.25 to 0.50, 0.50 to 0.75, and 0.75 to 1, all of which would last 1 year. In Version 1C, people were asked to value an extension of life of 1 year in QoL 0.25, of 6 months in QoL 0.5, and 4 months in QoL 0.75. By using these sub-versions, we could not only investigate whether changes in length and quality of life are valued differently, but also test whether only asking to value QoL or length of life differences would lead to different values than when both are valued within one questionnaire. We added these subversions to Version 1 since this would provide most valuable information in relation to our main research question (the extent to which elements beyond health are captured in current thresholds in CEAs and the impact thereof), given that current studies generally do not specifically ask for inclusion of elements beyond health. For all versions and scenarios, the scenario was constructed in such a way that respondents were asked to assume that they would die after the health change during the given period, to be able to compare the outcomes for different scenarios.

We asked respondents after the WTP tasks if they considered whether and how changing health would affect some specific elements beyond health when answering the WTP questions. These elements were utility of consumption, leisure, productivity, and spending patterns. For Version 2, we did not ask them whether they considered UoC since we explicitly instructed them to do so in the tasks. If people stated they considered a specific element, we further asked for their expectations regarding this element if their QoL would decrease to a worse QoL. For instance, if people stated they considered UoC, we asked whether they expected higher, lower, equal, or other UoC if their QoL would decrease. When the option ‘other’ was chosen, people could explain their expectations in an open answer format. For sub-versions 1A, 1C, and 2A, we additionally asked what they expected for an extension of life. For people considering an element, we furthermore asked to what extent they expected these considerations to have affected their WTP. When people stated they did not consider an element, we asked them to explain why not. For Version 1, we introduced people to the UoC scale at this point and asked to state their own UoC and the UoC in different health states as was done for Version 2 before the WTP tasks.

For both versions, we next asked people for their background characteristics such as age, gender, income, religion, and educational level. Afterwards, people were asked to evaluate the questionnaire on clarity of the different elements. To prevent answer option order bias all answers to multiple-choice questions were randomized for questions that did not have an ordinal order. To limit question order bias the different health states and changes in health states respondents were asked to evaluate were presented in a random order. We aimed to reduce hypothetical bias (the difference between stated and real values) by reminding people to consider current income. The questionnaire was pilot tested to determine the plausibility and clarity of the tasks, scales, and follow-up questions. In general, this showed the questionnaire was clear and feasible. Some respondents indicated the questionnaire would benefit from additional examples next to the UoC scale, which were added accordingly. The evaluation questions in the actual questionnaire showed this improved the clarity of the questionnaire.

### Statistical analyses

#### Exclusion criteria

For the main analysis, we excluded people who filled in the questionnaire too quickly (‘speeders’) or gave one or more protest answers. Whether respondents filled in the questionnaire too quickly was based on a time test for three researchers not related to this study. We asked them to complete the questionnaire quickly but still carefully, to obtain an indication of the time needed to fill in the questionnaire appropriately. The lowest time (9 min) was used as minimum. Whether answers were protest answers was based on the reason for stating a WTP of zero. From the options: (1) I cannot afford more, (2) medicines are not worth more than €0 to me, (3) medicines are worth more than €0 to me, but I rather spend the money differently, (4) I would rather not use medicines, (5) healthcare should be funded from the basic benefit package, (6) the value of health and healthcare cannot be expressed monetarily, or (7) other, option 5 and 6 options were considered protest answers. Open answers (option 7) were not classified as protest answers after inspection, as respondents mainly stated that the gain was too small to be worthwhile or the quality of life too low for life to be worth living.

#### Utility of consumption

Linear regression models were estimated to model respondents own experienced utility of consumption at time of the questionnaire as a function of respondent background characteristics such as age, income, and quality of life, which have previously shown or are expected to have impact on utility of consumption. Based on the questions in which we asked people to imagine what their utility of consumption would be for different qualities of life (25, 50, 75, and 100 on a 0–100 scale), we estimated a fixed effects model to model this utility of consumption as a function of quality of life. The fixed effects model allowed us to take into account unobserved factors (e.g., personality characteristics) that might influence an individual’s utility of consumption as the outcome variable consisted of repeated measurements of UoC within individuals.

#### Willingness to pay

We multiplied raw WTP values by 48 (by 4 as questions were based on a 0.25 QALY gain and by 12 as payment consisted of 12 monthly installments) to transform responses into WTP per QALY estimates. We compared the mean WTP values for respondents based on differences in in/exclusion of elements per scenario in which the QALY gain occurred and for the average WTP per respondent over the different scenarios using T-tests. In addition, we estimated a linear regression model where we modelled WTP per QALY as a function of the different experimental conditions as well as characteristics of the respondent. More specifically, we used age, gender, income, quality of life, utility of consumption, variables stating whether respondents spontaneously considered utility of consumption, leisure, productivity, and spending in addition to the dummies for sub-version of the questionnaire as explanatory variables. The WTP value we used as outcome in this regression was the mean WTP of each respondent calculated from the different scenarios in which the improvement in health was varied. We took the mean as a repeated measures ANOVA with Bonferroni correction showed no overall significant differences in the WTP values reported by the respondents for the different scenarios in which the improvement in health was varied.

#### Sensitivity analysis

We tested the robustness of our findings with a sensitivity analysis in which we present the outcomes of the main analyses in the paper executed both before and after excluding the respondents based on the predefined exclusion criteria. This includes an overview of the elements beyond health considered by the respondents during the WTP exercises and the outcomes of the regression analyses to estimate utility of consumption and the WTP for changes in health.

## Results

### Descriptives

After exclusion of speeders (n = 238) and people who gave one or more protest zero WTP (n = 37) the sample consisted of 890 respondents (6 people met more than 1 criterium). The characteristics of this sample are described in Table 1. Information is provided both for all versions combined and for the (sub)versions separately.

**Table 1:** Sample characteristics (n = 890)

	All versions (n = 890)	Version 1A (n = 214)	Version 1B (n = 239)	Version 1C (n = 230)	Version 2A (n = 207)
Age [years] (SD)	51.4 (15.9)	52.3 (15.2)	50.5 (16.6)	50.6 (16.2)	52.5 (15.4)
Gender [female]	53%	52.30%	54%	54.30%	51.20%
Educational level^a^					
Low	9.7%	10.70%	10.00%	8.70%	9.2%
Medium	51.8%	48.10%	49.00%	57.40%	52.7%
High	38.0%	40.70%	39.70%	33.50%	38.2%
Other	0.6%	0.50%	1.30%	0.40%	0.0%
Household income after tax (SD)	€3820 (8326)	€2690 (3399)	€3915 (8181)	€4462 (10,003)	€4165 (9835)
≤ €1999	34.6%	40.20%	30.10%	34.30%	34.3%
€2000–€3999	43.1%	42.10%	44.40%	42.60%	43.5%
≥ €4000	22.2%	17.80%	25.50%	23.00%	22.2%
Children [yes]	60.8%	61.70%	54.40%	66.50%	60.9%
Quality of life [0–100] (SD)	77.1 (16.5)	77.5 (16)	74.6 (16.4)	77.4 (17.2)	79 (16.3)
Utility of consumption [0–100] (SD)	76.9 (16.2)	76.3 (16.6)	74.4 (15.5)	75.6 (17.1)	81.8 (14.5)
Completion time [min] (SD)	32 (141.7)	21.4 (17.4)	38.5 (175.5)	33.5 (178.8)	33.7 (122.9)

Speeders (n = 238) and those with a protest zero WTP (n = 37) are excluded from this Table. 6 people met > 1 criterium

^a^Low = lower vocational and primary school, Medium = middle vocational and secondary school, High = higher vocational and academic education

### Elements beyond health

Table 2 shows the percentages of people spontaneously including (considering) an element beyond health in the evaluation tasks (WTP exercises). It further shows the expectations about the different elements for people who stated they considered these elements in their valuations. 30% of the respondents without instruction to do so (Version 1) considered utility of consumption. Of all respondents, 31% spontaneously considered leisure time, 22% considered productivity, and 52% considered spending without instruction to do so. Of the respondents who did not receive instruction to consider utility of consumption (Version 1), 18% considered no elements beyond health, 46% considered one element, 26% considered two elements, 7% considered three elements, and 4% considered all four elements beyond health. Of the respondents who did receive instruction to consider utility of consumption (Version 2), 21% considered no additional elements beyond health (next to UoC), 55% considered one additional element, 17% considered two additional elements, and 7% considered three additional elements beyond health. For people spontaneously considering utility of consumption, the largest share (48%) expected lower utility for lower quality of life and lower utility of consumption in additional length of life (42%). For leisure, the largest group (53%) expected less leisure in health states with lower quality of life but equal leisure in additional length of life (41%). 75% of those considering productivity expected lower productivity in worse health states and 50% expected lower productivity in additional length of life. For spending, the expectations were mixed, with 28% expecting equal, 24% less, and 29% more spending when experiencing lower quality of life. For additional lifetime, 35% expected equal, 22% less, and 28% expected more spending. For all elements, most people who included the element stated they expected their inclusion would only have little impact on their monetary valuations of health. For all elements, most people mentioned ‘the element had not crossed their mind’ as reason not to consider the element during the valuation.

**Table 2:** Information on the inclusion of elements beyond health in willingness to pay exercises

Elements (↓)	% Spontaneously including the element (%)	Expectations for lower quality of life					Expectations for additional lifetime				
		Equal (%)	Less/lower (%)	More/higher (%)	Different (%)	Other (%)	Equal (%)	Less/lower (%)	More/higher (%)	Different (%)	Other (%)
Utility of consumption^a^	30	24	48	27	–	2	32	42	25	–	2
Leisure^b^	31	29	53	16	–	1	41	35	23	–	0
Productivity^b^	22	22	75	2	–	1	37	50	13	–	0
Spending^b^	52	28	24	28	19^c^	2	35	22	28	14	0

Percentages spontaneously including elements and expectations on elements for changing health in terms of quality and length of life

^a^This includes only respondents who did not receive instruction to consider utility of consumption during the valuation (i.e., Version 1, n = 683)

^b^This includes both respondents who did (i.e., Version 2, n = 207) and respondents who did not (i.e., Version 1, n = 683) receive instruction to consider utility of consumption (i.e., Version 1 and 2, n = 890)

^c^Different implies total spending would remain equal but the distribution among different spending categories would be different

### Utility of consumption

The results from the regression analyses to estimate utility of consumption as function of quality of life (and other variables) are shown in Table 3. The first model is the multiple linear regression model of the respondent’s own utility of consumption at time of the questionnaire as function of health-related quality of life and respondent background characteristics. This model shows a significant positive relation between quality of life and utility of consumption (all described outcomes are ceteris paribus). That is, on average, people experience more utility from consumption when they experience better health-related quality of life. It also shows a significant negative association between being female or having children and utility of consumption. Model 2 in Table 3 shows the results from the fixed effects model in which we estimated the expected utility of consumption of the respondent for different health states. This model as well shows a significant positive association between quality of life and utility of consumption. By dividing the coefficients in this model by the change in QoL to which the coefficient is related, we can derive an average expected change in UoC from improving QoL with 1 unit of QoL (on the scale from 0 to 100). This reveals that the average change in utility of consumption per unit of quality of life decreases with the height of the quality of life in which the change occurs: a change from QoL 25 to QoL 50 is associated with an average increase in UoC of 0.39 per unit QoL; a change from QoL 25 to QoL 75 with an average increase of 0.35 per unit QoL; and a change from QoL 25 to QoL 100 with an average increase of 0.30 per unit QoL. Although somewhat lower, the magnitude of these changes is comparable to the changes reported in Model 1.

**Table 3:** Results regression analyses to estimate (respondent and expected) utility of consumption

	Dependent variable	
	(1) Respondent utility of consumption at time of questionnaire (SE) using ordinary least squares regression	(2) Expected utility of consumption for different health states (SE) using fixed effects regression
Quality of life (scale 0–100)	0.46*** (0.03)	
Quality of life (reference = 25)		
50		9.87*** (0.80)
75		17.48*** (0.80)
100		22.87*** (0.80)
Gender (female)	− 2.28** (0.96)	
Age	0.02 (0.03)	
Children (yes)	− 2.41** (1.07)	
Net household income	− 0.00 (0.00)	
Constant	42.88*** (3.24)	
Observations	890	3560
R^2^	0.24	0.26
Adjusted R^2^	0.23	0.01
Residual std. error	14.19 (*df* = 884)	
F Statistic	55.34*** (*df* = 5; 884)	331.01*** (*df* = 3; 2667)

*p < 0.1; **p < 0.05; ***p < 0.01

### Willingness to pay

Table 4 shows the mean WTP per QALY by scenario in which the health gain occurred and the mean of the average WTP per QALY per respondent (combining all scenarios) by groups (spontaneously) including or excluding the different elements beyond health in the evaluation. It also shows the results from tests for equality of means for groups instructed or not instructed to consider utility of consumption and for groups spontaneously including or excluding the elements utility of consumption, leisure time, productivity, and spending. Table 4 reports an overall mean WTP over all scenarios of €13,201 per QALY. No significant difference in WTP was found between respondents who were instructed and those who were not instructed to consider utility of consumption during the valuation. Table 4 also shows systematically higher although nonsignificant difference in WTP between people who spontaneously consider utility of consumption and those who do not, except for the scenario in which quality of life is improved from 25 to 50 for 1 year. For that scenario, the average WTP for people spontaneously including the element is €15,920, compared to €11,367 for people who do not (*p* = 0.05). No significant difference was found between the WTP for people who did and did not spontaneously consider leisure time in their valuation. The same holds for spontaneous inclusion of productivity, except for two scenarios. For the scenario in which quality of life was improved from 50 to 75 for 1 year, the average WTP is €19,169 for those who did and €12,418 for those who did not consider productivity (*p* < 0.10). For the scenario in which life was extended for 1 year in quality of 25, the average WTP was €6887 for those who did and €9988 for those who do not consider productivity (*p* < 0.05). Significant differences in WTP per QALY estimates were found between respondents who did and those that did not spontaneously consider spending during their valuation in half of the scenarios. In those scenarios WTP per QALY estimates were consistently found to be lower for respondents who spontaneously considered spending. The average mean WTP across all scenarios per respondent was also significantly different for people who considered spending compared to those who did not (€11,162 compared to €15,421, *p* = 0.05).

**Table 4:** Mean willingness to pay per quality-adjusted life-year

Element (↓)	Scenario ( →)	QoL 25 → 50, 1 year	QoL 50 → 75, 1 year^a^	QoL 75 → 100, 1 year	+ 1 year, QoL 25	+ 6 months, QoL 50^a^	+ 4 months, QoL 75	Mean all scenarios
Utility of consumption	No instruction to include^b^	12,876	13,943	15,869	8928	11,979	15,270	13,298
	Instruction to include	11,794	*–*	14,514	10,576	*–*	14,604	12,880
	*P*-value^c^	0.69	–	0.72	0.55	–	0.86	0.85
	No spontaneous inclusion	11,367	12,770	15,354	8075	10,672	14,536	12,347
	Spontaneous inclusion	15,920	16,459	17,061	10,904	15,443	17,242	15,500
	*P*-value	0.05^*^	0.22	0.70	0.22	0.20	0.52	0.17
Leisure time	No spontaneous inclusion	12,546	13,562	15,556	9139	10,432	15,556	13,198
	Spontaneous inclusion	12,847	14,922	15,561	9715	14,825	14,301	13,207
	*P*-value	0.88	0.65	1.00	0.77	0.16	0.70	1.00
Productivity	No spontaneous inclusion	12,299	12,418	15,232	9988	11,773	15,388	13,170
	Spontaneous inclusion	13,734	19,169	16,687	6887	12,780	14,095	13,309
	*P*-value	0.52	0.10^*^	0.68	0.04^**^	0.77	0.72	0.94
Spending	No spontaneous inclusion	13,796	16,690	19,628	8837	11,066	18,386	15,421
	Spontaneous inclusion	11,680	11,601	11,897	9764	13,011	11,706	11,162
	*P*-value	0.31	0.06^*^	0.05^*^	0.64	0.47	0.07^*^	0.05^*^
Overall mean		12,627	13,943	15,557	9315	11,979	15,114	13,201

By scenario and by respondents who were instructed to include or did/did not spontaneously include different elements beyond health

^a^This scenario was not included in Version 2 of the questionnaire (version with instruction to include utility of consumption)

^b^Includes both those who do and do not spontaneously included utility of consumption

^c^Comparison means of two above rows using Wilcoxon rank sum. Significances shown in asterisks: *=10%, **=5%, ***=1%

The results from the regression model to estimate the average willingness to pay per QALY per respondent as function of sub-version of the questionnaire, the inclusion of the elements beyond health, and background characteristics are shown in Table 5. This model shows no significant relation between sub-version of the questionnaire and WTP (all described outcomes are ceteris paribus). For all elements but spending, the spontaneous or instructed inclusion of the element was also not associated with significantly different WTP values. On average, respondents spontaneously considering the element spending reported a significantly (*p* < 0.05) lower WTP. Further, it shows a positive association between the respondent’s current utility of consumption and WTP. That is, on average, respondents who experience a higher utility of consumption are willing to pay more for an improvement in health (€200.1 per unit UoC on the scale from 0 to 100, *p* < 0.01). Also, females and older people report a significantly lower average WTP. People with higher incomes report a significantly higher average WTP, just as respondents who have a high educational level.

**Table 5:** Results regression analysis to estimate the willingness to pay per quality-adjusted life-year

	Dependent variable
	Mean willingness to pay (SE)
Sub-version questionnaire (reference = B: changes quality of life):	
A: changes quality and length of life	595.2 (2838.6)
C: changes length of life	− 2280.8 (2801.4)
Inclusion elements beyond health (reference = no inclusion element):	
Utility of consumption (spontaneous)	1135.6 (2563.4)
Utility of consumption (instructed)	− 2113.6 (3068.3)
Leisure (spontaneous)	− 1832.5 (2230.5)
Productivity (spontaneous)	− 2235.9 (2542.2)
Spending (spontaneous)	− 4365.9** (2050.4)
Quality of life	− 99.4 (70.3)
Utility of consumption	200.1*** (72.6)
Gender (female)	− 4307.4** (2058.2)
Age	− 144.0* (73.8)
Children (yes)	− 1372.6 (2299.7)
Net household income	0.4*** (0.1)
Educational level (reference = low):	
Medium	3073.0 (3598.7)
High	10,135.3*** (3759.4)
Other	1119.6 (13,885.2)
Constant	12,569.9 (8265.7)
Observations	890
R^2^	0.06
Adjusted R^2^	0.04
Residual std. error	29,969.22 (*df* = 873)
F Statistic	3.55 (*df* = 16; 873)

*p < 0.1; **p < 0.05; ***p < 0.01

### Sensitivity analysis

The section “Sensitivity analysis” in the appendix contains the full report of the results from the sensitivity analysis in which we present the outcomes of the main analyses in the paper executed both before and after excluding the respondents based on the predefined exclusion criteria. It shows consistency in the main findings from our results in relation to our research question ((i) finding that many people consider elements beyond health when valuing improvements in health in monetary terms, (ii) finding a positive association between health and utility of consumption, and (iii) finding no significant association between including elements beyond health and the height of the WTP (except for the element ‘spending patterns’)), supporting the robustness of our findings.

## Discussion and conclusion

In this paper we investigated the extent to which respondents consider elements beyond health when they are asked to assign a monetary value to improvements in health measured using QALYs. Research into this matter is important since it is currently unknown to what extent benefits beyond health are captured in cost-effectiveness analyses. We found that more than 80% of our sample considered elements beyond health when assigning monetary values to health states. Almost 80% considered additional elements beyond health (next to utility of consumption) when instructed to consider the element utility of consumption. We found that, without instruction to do so, still 30% considered utility of consumption, 31% considered leisure time, 22% considered productivity, and 52% considered spending patterns when answering questions on their willingness to pay for improvements in health. When asked about their expectations for these elements for changing health, answers varied. However, most people expected less/lower utility of consumption, leisure time, and productivity in lower quality of life. For spending, expectations were more evenly distributed among less, more, equal, and different distribution among categories. In addition, we found that utility of consumption is expected to increase with quality of life. Although all these findings suggests that the WTP for a QALY covers more than health alone, the impact of the inclusion of non-health elements on the monetary value of a QALY appears to be limited in our results. While for some elements (including utility of consumption) spontaneous inclusion appeared to increase WTP values in most scenarios, the difference was rarely significant. Also in a regression analysis, only the spontaneous inclusion of spending during the valuation was associated with a significantly different (lower) average WTP per respondent when compared to respondents who did not consider the element in the current study.

An important strength of this study is that we are the first to provide more insight into the elements beyond health considered in the monetary valuation of the QALY through WTP. Although comparable research has been conducted in the area of assigning QALY weights to descriptive health states, to our knowledge no such research was previously performed in the context of the monetary value for a QALY. The issue of elements considered in the CEA framework in general has been discussed frequently but not yet in an empirical and direct approach. Another strength of this study is that we measure health state dependency in a new and direct manner by introducing the utility of consumption scale and asked respondents about their own and their expected utility of consumption in different health states. Our results suggest health state dependency of utility of consumption, which, if shown to be true, has important implications.

There are also some noteworthy limitations of our study. First, the scenarios presented to the respondents are not fully compatible with how healthcare is organized in the Netherlands. Although we asked respondents for their WTP for healthcare, most healthcare in the Netherlands is publicly organized and people generally only pay a limited amount directly related to healthcare consumption in 1 year. This adds to the already existing general hypothetical bias from using hypothetical scenarios rather than actual decision making in this context. However, although this bias presumably impacts the valuation of respondents, this is arguably less relevant in relation to our current research question on the elements considered in the WTP. Another limitation of our research is that directly asking respondents whether they considered elements beyond health may have framed their answering to elements considered. For instance, answering might have been different when we would have asked more generally and in an open format about what people considered. However, the current format enabled us to obtain information specifically about relevant elements in a structured way, allowing testing for the influence of their inclusion, and arguably helped respondents in answering the questions. Similarly, while our explanation of the potential relationship between health and the utility of consumption was intended to help respondents understand the concept—based on feedback from the pilot questionnaire—it may have inadvertently influenced their responses regarding this relationship. Another issue worth noting is that the questionnaire was administered during the COVID-19 pandemic. During this period, people were limited in their freedom to live their lives the way they preferred due to measures installed by the Dutch government (e.g., the partial lockdown) to reduce the outbreak and consequences of infections. The state of the world at time of the questionnaire might have affected the answers of the respondents in several ways. For instance, due to the limitations, more people might have thought about issues beyond health when valuing health due to increased awareness of the broader impact of illness on life and the value of health itself might have been affected as well. To obtain information on the actual impact of such potential effects, it would be needed to repeat the experiment (now the COVID-19 pandemic is no longer current). A limitation related to the utility of consumption scale is that it may be considered to be too simple, and its validity has not been established. In using it jointly with a VAS for health, we hoped to achieve consistency in how elements in the questionnaire were illustrated and measured to respondents, and to lower the cognitive burden on respondents. The current approach was considered reasonable for gaining first insights into utility of consumption in relation to health states in the context of the WTP for a QALY, especially given that previous attempts only estimated utility of consumption in an indirect manner [16, 23]. Also, the pilot and evaluation questions as well as the way in which respondents reflected on the concepts indicated a proper understanding of the concept. However, more research is needed into the accuracy of this direct UoC measure and also on how the presentation of the scale and scenario’s in general could be improved. Specifically mentioning ‘health-related quality of life’ rather than ‘quality of life’ when using the VAS scale and describing the improvements in health might, for example, further clarify the distinction between both concepts. A final limitation is that there are differences in income between the groups in the different subversions, which points to the broader issue that there may be an imbalance in potential unobserved confounders. Although we controlled for income in our statistical analyses, unobservable confounders could still have introduced bias into our estimates.

There are different types of research to which elements of our research can be compared. First, is research into elements beyond health considered in the process of assigning QALY weights to descriptive health states. In one of these studies, it was found that 40% spontaneously considered the impact of ill-health on income while 75% spontaneously considered the impact on leisure when valuing health states on a VAS scale. However, spontaneous inclusion was not associated with different valuations, whereas explicit instruction to include income was associated with lower valuations [24]. In another example in the context of assigning QALY weights, 36% of the respondents spontaneously considered income and 84% spontaneously considered the effects of ill health on leisure activities when they were not instructed to do so. Furthermore, neither explicit instruction to consider the influence of ill health on income nor spontaneous consideration of the influence on income or seemed to be related to differences in health state valuations [20]. Others found that explicit instruction to consider the utility of consumption and leisure did not lead to significantly different valuations in time-trade-off exercises, which are used to convert general descriptions of health states into utility values (used to calculate QALYs). However, spontaneous consideration (which happened in 60% of the respondents who did not receive explicit instructions) of those elements was associated with lower health state valuations [11]. Unfortunately, in that study the inclusion of both elements was considered simultaneously, limiting insight into the separate elements. In general, the outcomes of our research are comparable with the outcomes of research in the area of assigning QALY weights to descriptive health-states. In both, it is found that many people consider elements beyond health next to health when valuing health states. Also in both, the impact of instructed or spontaneous inclusion of elements beyond health on the outcome (here WTP per QALY) appears to be limited. When comparing the findings in more detail, some differences can be found as well. For instance, respondents were generally less likely to consider leisure (31%, compared to 75%, 84%, and 60%) or utility of consumption (60%, compared to 30%) in our research focusing on the monetary valuation of a QALY than those in studies investigating health state valuations. This suggests that the context of studies may matter in what people consider, and, therefore, that there may be differences between elements captured or considered in the QALY and elements captured or considered in the WTP for a QALY, raising questions about consistency. It is important to further research these potential differences as these may complicate the consistency and interpretation of the CEA framework. Even more so since our study highlights that people expect differences in these elements for different health-states.

Other research to compare with our findings, is research into the WTP for a QALY from an individual perspective. Earlier, comparable research on WTP using VAS health-states for the Netherlands found a mean WTP per QALY of €12,900 [12]. This mean WTP is quite comparable to the mean WTP of €13,134 found in our study. Although several estimates of WTP per QALY values exist, it is difficult to make further meaningful comparisons, given the wide range of differences in the methodologies. For instance, a review of WTP per QALY values showed values ranging from €1000 to €4,800,000 per QALY [14]. Moreover, the emphasis in our study was more on elements included in the monetary valuation of QALYs than in the valuation itself. Arguably, other approaches (e.g., valuing more marginal health changes under uncertainty) may be more appropriate for that. Finally, the outcomes of our research can also be compared to research into health state dependency of utility of consumption. Previous research showed mixed evidence regarding the existence as well as the direction of health state dependency [17, 18]. Our results suggest that health state dependency at least is expected to exist in our respondents and that they expect the utility of consumption to increase with health improvements, but at a decreasing rate.

There are several important implications of our results. First, the finding that the impact of stated inclusion of elements beyond health on the value of a QALY was limited, suggests that these benefits are not fully and consistently captured in this value of the QALY. For adequate valuation of these elements to be the case, it would be expected that the consideration of these elements by respondents would (at least) have affected their value of the QALY. (Note that although this explanation appears reasonable, other explanations can be found as well, e.g., the limited impact could imply that people attach relatively little value to these elements when compared to health.) This would also suggest that (stated) consideration of elements does not imply (adequate) valuation of elements, even when specific information on the element and instructions to consider the implications thereof are provided. If the aim of CEA from a societal perspective would be to consistently capture all costs and all benefits, this raises the need for other (explicit) methods and/or instructions to capture benefits beyond health in future CEA in a comprehensive and consistent manner, both in terms of outcomes and their valuation. If these would be included in addition to QALYs, the risk of double counting the beyond health elements seems negligible given our results.

A more fundamental implication of our findings is that it is difficult to reconcile current CEA with conventional cost–benefit analysis. If, like our results indicate, the utility of consumption depends on the health state people are in, this violates a key assumption typically used to establish a welfare theoretic underpinning of CEA [19]. These implications together raise important questions for the practice of CEA as other normative underpinnings for current practice may then be needed (if current practice is seen as desirable). We argue that given that health and non-health benefits depend on each other and are difficult to isolate a societal perspective still is warranted. A more pragmatic underpinning of this could be one in which a societal decision maker wants to maximize health given societal resources. (Note that one might argue that such an approach comes with own issues and inconsistencies.) Given the special role of health and health spending, we recommend that, at least for now, results from a narrower healthcare perspective should be presented alongside results from a societal perspective (4).

The finding that elements beyond health are affected by health state also has implications for the methods for the estimation of these costs. For the Netherlands, for instance, standardized estimates of these costs have been developed (which can be presented alongside currently required elements of CEA to provide a more comprehensive view of the impact of investing in healthcare interventions for society). These standardized estimates of non-medical consumption are averages by age but not by health status. If utility of consumption and spending are expected to be affected by health, this should presumably also be captured in the estimates of these costs. This also shows that non-medical consumption is not only relevant for life-years gained from interventions, but also for life-years lived with and without the intervention. Inclusion of non-medical costs in economic evaluation may therefore be important not only for life-extending interventions, as part of total future costs, but also for quality of life improving interventions.

Several suggestions for further research can be made in addition to those already mentioned above. For instance, qualitative research can be performed to gain more insight into the cognitive processes and reasons for why people consider some elements in valuing health and what exactly it is that they value (and based on which expectations). This could also provide different and more detailed evidence on the extent to which people consider elements beyond health (in addition to the questions we asked). For this, think out loud studies could be used, or in-depth interviews around some of the questions used in our questionnaire. Moreover, in our WTP study we applied the individual perspective. Comparable research can also be performed from other perspectives, such as the societal or the socially inclusive perspective. It may be that other elements are considered in such a context, and the impact of inclusion may be different when valuing changes in health in other persons rather than in oneself. Additional research could also vary how the changes in health are presented to the respondent. While in this research a VAS scale was used to describe health status, other ways, like using the EQ-5D descriptive system may harmonize the elements of health considered. More specific descriptions of health states (such as in the EQ-5D) may also result in different considerations of elements beyond health (e.g., through different associations). Also, different sizes of health gains and different contexts which may be closer to reality may provide different insights, albeit that becoming more specific in describing treatments and outcomes limits the generalizability of the setting and results. As already mentioned above, it is also important to establish the validity of the utility of consumption scale that was used in this research and compare our methods and findings in more detail with comparable previous and future approaches.

Concluding, we find that many respondents consider elements beyond health when valuing QALYs but that the impact of the inclusion of these elements on the monetary value of a QALY is limited. Our findings therefore illustrate that it is difficult to isolate health benefits from non-health benefits and to consistently capture these in economic evaluations. They also suggest that reconciling current CEA with welfare economics remains challenging and may require other normative models to guide CEA conducted from a societal perspective and/or improved methods for performing CEA.

## Electronic supplementary material

Below are the links to the electronic supplementary material. This contains (information about) the questionnaire and the data, which can also be accessed via the EUR Data Repository, 10.25397/eur.24926226.

Supplementary file1 (DOCX 12 kb)

Supplementary file2 (DOCX 14 kb)

Supplementary file3 (XLSX 45 kb)

Supplementary file4 (DOCX 105 kb)

Supplementary file5 (DOCX 103 kb)

Supplementary file6 (DOCX 111 kb)

Supplementary file7 (DOCX 238 kb)

Supplementary file8 (SAV 3410 kb)

Supplementary file9 (SAV 4600 kb)

Supplementary file10 (SAV 4533 kb)

Supplementary file11 (SAV 4094 kb)

Supplementary file12 (XLSX 810 kb)

Supplementary file13 (R 51 kb)

## Data Availability

The datasets generated by the survey research and analyzed during the current study are available in the EUR Data Repository, 10.25397/eur.24926226.

## Appendix

### Examples questionnaire

This appendix shows examples from the questionnaire administered for this research. Note that the examples in this appendix are translated from Dutch to English. The original questionnaire was administered in Dutch. The complete questionnaire is available in the EUR Data Repository, 10.25397/eur.24926226.

Note that the dataset in the repository includes the questionnaire in Word format. Respondents had access to an online link to a digitalized version of the questionnaire during the administering of the questionnaire (the links to the digitalized versions of the questionnaire could not be publicly provided by the sampling agency).

Also note that the numbering of the subversions in the repository (1–4) is different to the numbering used in this article (1A–2A). These translate as follows: Version 1 in the dataset in the repository = Version 1B in this article, Version 2 in the dataset = Version 1C in this article, Version 3 in the dataset = Version 1A in this article, and Version 4 in the dataset = Version 2A in this article.

#### Introduction utility of consumption and utility of consumption scale

Your utility of consumption may differ in different health conditions. With utility of consumption, we mean the satisfaction you experience from purchasing and consuming goods and services.

For example, you may enjoy things such as food, sports, hobbies, going out, or holidays less if your health is poorer and therefore experience less satisfaction. At the same time, you could obtain more fulfillment from more practical services such as domestic help, which will increase your satisfaction. With utility of consumption, we refer to your overall satisfaction with your total consumption.

To express utility of consumption we use a utility of consumption scale. The figure below shows the utility of consumption scale. You can think of this as a utility thermometer with 'no utility' at the bottom (score 0) and 'highest utility achievable' at the top (score 100).

#### WTP scenario Version 1

Imagine that due to illness you have 1 year left to live in a quality of life of 25 on the scale of 0 (dead) to 100 (completely healthy). A drug can increase your quality of life from 25 to 50 this year.

The figure below shows this situation and the increase in quality of life due to this drug.

For this increase in quality of life, you must take the drug (without side effects) every month for a year. Your quality of life will certainly increase from 25 to 50. You must pay for the medicine by yourself, from your own (family) income. Payment is made in 12 monthly installments. Your income is equal to your current income throughout the entire period.

Would you like to go through the amounts below, from low to high, and choose the highest amount that you would certainly be willing to pay per month for this drug? (for example: if you are sure you would like to pay €100 a month for this drug, but not sure if you would pay €150 a month for it, choose €100).

**Table:**

€0	€10	€15	€25	€50	€75	€100	€125	€150	€200	€250	€300	€500	€750	€1000	€1500	€2500	…
O	O	O	O	O	O	O	O	O	O	O	O	O	O	O	O	O	O

#### WTP scenario Version 2

Imagine that due to illness you have 1 year left to live in a quality of life of 25 on the scale of 0 (dead) to 100 (completely healthy). A drug can increase your quality of life from 25 to 50 in this year. The left figure below shows this situation and the increase in quality of life due to this drug.

In the quality of life of 25, you have a utility of consumption of 34^3^ on the scale of 0 (no utility) to 100 (highest achievable utility). In the quality of life of 50, you have a utility of consumption of 66. The right figure below shows the increase in the utility of consumption due to the increase in quality of life caused by this drug.

For this increase in quality of life, you must take the drug (without side effects) every month for a year. Your quality of life will certainly rise from 25 to 50 and your utility of consumption from 34 to 66. You must pay for the medicine by yourself, from your own (family) income. Payment is made in 12 monthly installments. Your income is equal to your current income throughout the entire period.

Would you like to go through the amounts below, from low to high, and choose the highest amount that you would certainly be willing to pay per month for this drug? (for example: if you are sure you would like to pay €100 a month for this drug, but not sure if you would pay €150 a month for it, choose €100). Consider the impact of quality of life on your utility of consumption.

**Table:**

€0	€10	€15	€25	€50	€75	€100	€125	€150	€200	€250	€300	€500	€750	€1000	€1500	€2500	…
O	O	O	O	O	O	O	O	O	O	O	O	O	O	O	O	O	O

### Sensitivity analysis

In this sensitivity analysis the main analyses in the paper are executed both before and after excluding respondents based on the predefined exclusion criteria. Table 6 shows the percentages of respondents considering elements beyond health and their expectations about these elements (based on Table 2 in the paper) before and after excluding respondents based on the predefined exclusion criteria.

**Table 6:** Sensitivity analysis based on Table 2 (Information on the inclusion of elements beyond health in willingness to pay exercises)

Elements (↓)	% Spontaneously including the element	*Expectations for lower quality of life*					*Expectations for additional lifetime*				
		Equal (%)	Less/lower (%)	More/higher (%)	Different (%)	Other (%)	Equal (%)	Less/lower (%)	More/higher (%)	Different (%)	Other (%)
*Before exclusion respondents*											
Utility of consumption^a^	29	25	50	23	–	2	34	43	22	–	1
Leisure^b^	31	29	55	15	–	1	38	39	22	–	0
Productivity^b^	23	24	71	4	–	0	35	52	13	–	0
Spending^b^	47	30	24	29	17^c^	1	35	26	26	13	0
*After exclusion respondents*											
Utility of consumption^d^	30	24	48	27	–	2	32	42	25	–	2
Leisure^e^	31	29	53	16	–	1	41	35	23	–	0
Productivity^e^	22	22	75	2	–	1	37	50	13	–	0
Spending^e^	52	28	24	28	19^c^	2	35	22	28	14	0

Percentages spontaneously including elements and expectations on elements for changing health in terms of quality and length of life. Shows outcomes both before and after excluding respondents based on exclusion criteria

^a^This includes only respondents who did not receive instruction to consider utility of consumption during the valuation (i.e., Version 1, n = 871)

^b^This includes both respondents who did (i.e., Version 2, n = 288) and respondents who did not (i.e., Version 1, n = 871) receive instruction to consider utility of consumption (i.e., Version 1 and 2, n = 1159)

^c^Different implies total spending would remain equal but the distribution among different spending categories would be different

^d^This includes only respondents who did not receive instruction to consider utility of consumption during the valuation (i.e., Version 1, n = 683)

^e^This includes both respondents who did (i.e., Version 2, n = 207) and respondents who did not (i.e., Version 1, n = 683) receive instruction to consider utility of consumption (i.e., Version 1 and 2, n = 890)

Table 7 shows the outcomes of the regression analyses to estimate (respondent and expected) utility of consumption (based on Table 3 in the paper) before and after excluding respondents based on the predefined exclusion criteria.

**Table 7:** Sensitivity analysis based on Table 3 (regression analyses to estimate (respondent and expected) utility of consumption)

	Before exclusion respondents		After exclusion respondents	
	Dependent variable		Dependent variable	
	(1) Respondent utility of consumption at time of questionnaire (std. error) using ordinary least squares regression	(2) Expected utility of consumption for different health states (std. error) using fixed effects regression	(1) Respondent utility of consumption at time of questionnaire (std. error) using ordinary least squares regression	(2) Expected utility of consumption for different health states (std. error) using fixed effects regression
Quality of life (scale 0–100)	0.49*** (0.03)		0.46*** (0.03)	
Quality of life (reference = 25):				
50		9.63*** (0.70)		9.87*** (0.80)
75		16.04*** (0.70)		17.48*** (0.80)
100		21.42*** (0.70)		22.87*** (0.80)
Gender (female)	− 1.17 (0.86)		− 2.28** (0.96)	
Age	0.03 (0.03)		0.02 (0.03)	
Children (yes)	− 1.90** (0.95)		− 2.41** (1.07)	
Net household income	− 0.00 (0.00)		− 0.00 (0.00)	
Constant	38.56*** (2.79)		42.88*** (3.24)	
Observations	1159	4636	890	3560
R^2	0.25	0.23	0.24	0.26
Adjusted R^2	0.25	− 0.03	0.23	0.01
Residual Std. Error	14.50 (*df* = 1153)		14.19 (*df* = 884)	
F Statistic	77.18*** (*df* = 5; 1153)	344.87*** (*df* = 3; 3474)	55.34*** (*df* = 5; 884)	331.01*** (*df* = 3; 2667)

Shows outcomes before and after excluding respondents based on exclusion criteria

**p* < 0.1; ***p* < 0.05; ****p* < 0.01

Table 8 shows the outcomes of the regression analysis to estimate the willingness to pay per quality-adjusted life-year (based on Table 5 in the paper) before and after excluding respondents based on the predefined exclusion criteria.

**Table 8:** Sensitivity analysis based on Table 5 (Results regression analysis to estimate the willingness to pay per quality-adjusted life-year)

	Before exclusion respondents	After exclusion respondents
	Dependent variable	Dependent variable
	Mean willingness to pay (std. error)	Mean willingness to pay (std. error)
Sub-version questionnaire (reference = B: changes quality of life):		
A: changes quality and length of life	1498.8 (2656.8)	595.2 (2838.6)
C: changes length of life	− 1542.8 (2627.5)	− 2280.8 (2801.4)
Inclusion elements beyond health (reference = no inclusion element):		
Utility of consumption (spontaneous)	1087.2 (2414.3)	1135.6 (2563.4)
Utility of consumption (instructed)	− 1487.0 (2762.5)	− 2113.6 (3068.3)
Leisure (spontaneous)	− 2383.3 (2042.5)	− 1832.5 (2230.5)
Productivity (spontaneous)	− 1.296.2 (2.2831)	− 2235.9 (2542.2)
Spending (spontaneous)	− 4753.6** (1880.6)	− 4365.9** (2050.4)
Quality of life	− 147.0** (64.8)	− 99.4 (70.3)
Utility of consumption	238.3*** (65.2)	200.1*** (72.6)
Gender (female)	− 3985.9** (1884.8)	− 4307.4** (2058.2)
Age	− 115.0* (66.4)	− 144.0* (73.8)
Children (yes)	− 1503.0 (2077.6)	− 1372.6 (2299.7)
Net household income	0.4*** (0.1)	0.4*** (0.1)
Educational level (reference = low)		
Medium	3460.0 (3344.5)	3073.0 (3598.7)
High	9748.7*** (3490.8)	10,135.3*** (3759.4)
Other	1647.9 (14,480.5)	1119.6 (13,885.2)
Constant	10,401.7 (7412.6)	12,569.9 (8265.7)
Observations	1159	890
R^2^	0.06	0.06
Adjusted R^2^	0.05	0.04
Residual std. error	31,491.0 (*df* = 1142)	29,969.22 (*df* = 873)
F statistic	4.6 (*df* = 16; 1142)	3.55 (*df* = 16; 873)

Shows outcomes before and after excluding respondents based on exclusion criteria

**p* < 0.1; ***p* < 0.05; ****p* < 0.01

## Footnotes

1 The questionnaire was administered during the COVID-19 pandemic. For the actual status of the pandemic in the Netherlands and related measures from the government at that point in time we refer to the COVID-19 information page from the Dutch government: https://www.rijksoverheid.nl/onderwerpen/coronavirus-tijdlijn/2020 (in Dutch).

2 The exact monetary value of these points could not be disclosed by the sampling agency but could not exceed the maximum value of €1 per questionnaire.
