# Supplementary material for: Benefits beyond health in the willingness to pay for a quality-adjusted life-year
Source: Eur J Health Econ. 2024 Oct 7;26(4):653–70. doi: 10.1007/s10198-024-01726-7 (PMC12126320; doi:10.1007/s10198-024-01726-7)
Supplement: Supplementary file 1 — Supplementary file1 (DOCX 12 kb) [file 10198_2024_1726_MOESM1_ESM.docx]

***Description accompanying the dataset (landing page for the link provided in the unblinded manuscript)***

**Benefits beyond Health in the Willingness to Pay for a Quality-Adjusted Life-Year**

This study investigates whether people consider elements beyond health when valuing Quality-Adjusted Life-Years (QALYs) monetarily and the influence of inclusion on this value. A Willingness to Pay (WTP) experiment was administered among the general public in which people were asked to assign monetary values to QALYs. Our results show that (stated) UoC increases with quality of life but that instructing people to consider UoC does not impact their monetary valuation of the QALY. Furthermore, many respondents consider elements beyond health when valuing QALYs but the impact on the monetary value of a QALY is limited.

This dataset includes the documents related to the construction of the (sub-versions of the) questionnaire, the raw data from the (subversions of the) questionnaire collected by and received from the sampling agency, and the data after merging the individual datasets for the subversions into one dataset, and the code to analyze the data.
