# Supplementary material for: Benefits beyond health in the willingness to pay for a quality-adjusted life-year
Source: Eur J Health Econ. 2024 Oct 7;26(4):653–70. doi: 10.1007/s10198-024-01726-7 (PMC12126320; doi:10.1007/s10198-024-01726-7)
Supplement: Supplementary file 2 — Supplementary file2 (DOCX 14 kb) [file 10198_2024_1726_MOESM2_ESM.docx]

**Description questionnaire and data collection – Benefits beyond Health in the Willingness to Pay for a Quality-Adjusted Life-Year**

We conducted a study into the Willingness to Pay (WTP) for a Quality Adjusted Life-Year (QALY) from an individual perspective. For this, we asked people for their WTP for health improvements in terms of quality of life or length of life. Depending on the version they were assigned to, people were asked to value changes in quality of life, length of life, or both. All changes in health resulted in a total QALY gain of 0.25. For the period of one year when these resulted from changes in quality of life and with varying length when these resulted from changes in length of life (length depending on the quality of life in which the gain occurred). Quality of life before the health gain varied between 0.25, 0.50 and 0.75.

We asked people to assume their income to be and remain equal to their own current income. Next to questions on respondent characteristics (age, gender, income, education, religion, etc.), we asked people after answering the WTP questions for the extent to which they had considered elements beyond health and for their expectations for these elements for changes in health. We focused on the elements production, leisure time, spending patterns, and utility of consumption. We also asked people for their own quality of life and utility of consumption. In one of the versions of the questionnaire, we asked people explicitly to consider utility of consumption when valuing the QALY gains.

During the questionnaire, we used the ‘utility of consumption scale’ to show how utility of consumption would change for changing health. This scale was constructed for the purpose of this research and is comparable to the EQ-VAS. It ranges from 0 (no utility) to 100 (highest utility achievable) instead of the 0 (worst imaginable health state) to 100 (best imaginable health state) of the EQ-VAS. For some versions of the questionnaire, we also asked people if and how they expected their utility of consumption would change when they would receive an amount of €100.000. We asked this for different levels of quality of life.

A total of 1.159 respondents were sampled to be representative of the Dutch general public by age (18–75 years), sex, and education level and to cover a broad range of household incomes. The survey was administered online by a professional research agency (Dynata) in November and December 2020.

Although the questionnaire was administered in Dutch, the document describing the questionnaire and dataset itself contains descriptions in English. This makes it possible for people who do not read/speak Dutch to understand the questionnaire and (most of the) data.
