# Supplementary material for: Benefits beyond health in the willingness to pay for a quality-adjusted life-year
Source: Eur J Health Econ. 2024 Oct 7;26(4):653–70. doi: 10.1007/s10198-024-01726-7 (PMC12126320; doi:10.1007/s10198-024-01726-7)
Supplement: Supplementary file 5 — Supplementary file5 (DOCX 103 kb) [file 10198_2024_1726_MOESM5_ESM.docx]

Version 2 – No instruction + Scenario set 2 (LL)

# A: Introduction and permission

## A.1 Introduction to the questionnaire and permission

Het budget voor gezondheidszorg is beperkt. Beleidsmakers moeten daarom keuzes maken over welke behandelingen vergoed kunnen worden via de basis zorgverzekering. Bij het maken van deze keuzes houden beleidsmakers rekening met de waarde die Nederlanders hechten aan zorg en gezondheid. Dit onderzoek gaat hierover.

Het onderzoek wordt uitgevoerd door de Erasmus Universiteit Rotterdam. In deze vragenlijst wordt u gevraagd op verschillende manieren bepaalde gezondheidstoestanden en veranderingen te beoordelen. Probeer u bij het beantwoorden van deze vragen zo goed mogelijk voor te stellen wat deze gezondheidstoestanden en veranderingen voor u zouden betekenen. Er zijn geen goede of foute antwoorden, we zijn geïnteresseerd in uw mening.

Deze vragenlijst bestaat uit vier delen en deelname duurt ongeveer 20 minuten. Het is belangrijk dat u alle vragen zorgvuldig leest en op iedere vraag antwoord geeft. Uw antwoorden worden volledig anoniem verwerkt.

*Begrijpt u het doel van dit onderzoek, wilt u aan dit onderzoek deelnemen en geeft u ons toestemming om uw antwoorden anoniem te gebruiken voor wetenschappelijk onderzoek?*

- Ja
- Nee

*U kunt uw deelname hierna op ieder gewenst moment alsnog beëindigen.*

## A.2 Permission received [text only if A.1 = ‘Ja’]

Fijn dat u deze vragenlijst wilt invullen. Alvast bedankt voor uw medewerking. De vragenlijst start in het volgende scherm.

## A.3 Permission not received [text only if A.1 = ‘Nee’ 🡪 for these people this is the last screen]

Jammer dat u deze vragenlijst niet wilt invullen. Dit is voor u het einde van dit onderzoek.

# B: Part 1 - Introduction scale(s) + tasks

Als voorbereiding op het eerste deel van de vragenlijst geven wij hierna enkele voorbeelden van de scenario’s die door de vragenlijst heen worden gebruikt. Te beginnen met de omschrijving van de gezondheidstoestanden.

## B.0 Introduction health utility scale

### B.0.A Introduction health utility scale I

In de vragenlijst worden verschillende gezondheidstoestanden beschreven. De *kwaliteit van leven* in de gezondheidstoestanden wordt uitgedrukt in een score tussen 0 en 100. De score 0 staat gelijk aan de dood. De score 100 staat gelijk aan de kwaliteit van leven in perfecte gezondheid.

Het kan bijvoorbeeld zo zijn dat u door een ziekte veel pijn heeft en u ernstig wordt belemmerd in uw dagelijks leven en zelfstandigheid, waardoor uw kwaliteit van leven relatief laag is. Een ander voorbeeld is dat u af en toe last heeft van hoofdpijn, maar dit over het algemeen geen grote impact heeft op uw leven. Dit zou passen bij een relatief hoge kwaliteit van leven.

Hieronder ziet u een figuur die de kwaliteit van leven schaal laat zien. U kunt dit zien als een gezondheidsthermometer met onderaan ‘dood’ (score 0) en bovenaan ‘volledig gezond’ (score 100).

### B.0.B Introduction health utility scale II (own health utility)

Hoe zou u uw huidige gezondheid beoordelen op de schaal van 0 (dood) tot 100 (perfecte gezondheid)?

Geef op de schaal in de figuur hieronder aan welk getal uw gezondheid het beste weergeeft. Dit doet u door op het bolletje te klikken en dit met uw muis ingedrukt naar boven of beneden te schuiven.

**< answer should be in range 0-100, answer should become visible on scale in figure below>**

### B.0.C Introduction health utility scale III

Naast *kwaliteit van leven* wordt in de vragenlijst ook de *lengte van leven* aangegeven. Stelt u zich voor dat u nog *één jaar* te leven heeft in een kwaliteit van leven van 100 op de schaal van 0 (dood) tot 100 (volledig gezond). Hieronder ziet u een figuur die deze situatie laat zien.

### B.0.D Introduction health utility scale IV

Stelt u zich voor dat u door een ziekte nog één jaar te leven heeft in een kwaliteit van leven van 80 op de schaal van 0 (dood) tot 100 (volledig gezond). Hieronder ziet u een figuur die deze situatie laat zien.

### B.0.E Introduction health utility scale V

In de vragenlijst worden verschillende veranderingen in gezondheidstoestanden beschreven als gevolg van het gebruik van een medicijn. In dit voorbeeld beschrijven wij een verandering in de *kwaliteit van leven* door een medicijn.

Stelt u zich voor dat u door een ziekte nog één jaar te leven heeft in een kwaliteit van leven van 80 op de schaal van 0 (dood) tot 100 (volledig gezond). Een medicijn kan ervoor zorgen dat uw kwaliteit van leven in dit jaar stijgt van 80 naar 100.

Hieronder ziet u een figuur die deze situatie en de stijging van de kwaliteit van leven door dit medicijn laat zien.

### B.0.F Introduction health utility scale VI

In dit voorbeeld beschrijven wij een verandering in de *lengte van leven* als gevolg van het gebruik van een medicijn.

Stelt u zich voor dat u door ziekte nog één jaar te leven heeft in een kwaliteit van leven van 80 op de schaal van 0 (dood) tot 100 (volledig gezond). Een medicijn kan ervoor zorgen dat u één jaar langer leeft in kwaliteit van leven van 80.

Hieronder ziet u een figuur die deze situatie en de levensverlenging door dit medicijn laat zien.

## B.1 WTP LL +25, 1 year

Dit was het laatste voorbeeld voor het eerste deel van de vragenlijst. Er worden hierna steeds enkele scenario’s beschreven die vergelijkbaar zijn met de scenario’s in de voorbeelden. Wij stellen u vervolgens enkele vragen over wat u zou willen betalen voor het medicijn dat wordt beschreven in deze scenario’s.

### B.1.A WTP changes length of life +25, 1 year

Stelt u zich voor dat u door ziekte nog één jaar te leven heeft in een kwaliteit van leven van 25 op de schaal van 0 (dood) tot 100 (volledig gezond). Een medicijn kan ervoor zorgen dat u één jaar langer leeft in kwaliteit van leven van 25.

Hieronder ziet u een figuur die deze situatie en de levensverlenging door dit medicijn laat zien.

Voor deze levensverlenging moet u het medicijn (zonder bijwerkingen) een jaar lang elke maand innemen. U leeft dan zeker één jaar langer in een kwaliteit van leven van 25. U moet het medicijn zelf betalen, uit uw eigen (gezins)inkomen. De betaling vindt plaats in 12 maandelijkse termijnen (u betaalt dus niet voor dit medicijn in de periode dat u langer leeft). Uw inkomen is in de gehele periode gelijk aan uw huidige inkomen.

Wilt u de onderstaande bedragen afgaan, van laag naar hoog, en het hoogste bedrag kiezen dat u zeker wel **per maand** zou willen betalen voor dit medicijn? (bijvoorbeeld: als u zeker bent dat u €100 per maand zou willen betalen voor dit medicijn, maar niet zeker of u er €150 per maand voor over zou hebben, kiest u €100).

| €0 | €10 | €15 | €25 | €50 | €75 | €100 | €125 | €150 | €200 | €250 | €300 | €500 | €750 | €1000 | €1500 | €2500 | … |
| --- | --- | --- | --- | --- | --- | --- | --- | --- | --- | --- | --- | --- | --- | --- | --- | --- | --- |
| O | O | O | O | O | O | O | O | O | O | O | O | O | O | O | O | O | O |

### B.1.B WTP changes length of life +25, 1 year

U heeft aangegeven dat u zeker 12 maanden [answer question B.1.A] per maand wilt betalen voor een medicijn dat zorgt dat u één jaar langer leeft in kwaliteit van leven van 25 op de schaal van 0 (dood) tot 100 (perfecte gezondheid).

Wilt u de onderstaande bedragen afgaan, van laag naar hoog, en het laagste bedrag kiezen dat u zeker niet **per maand** zou willen betalen voor dit medicijn? (bijvoorbeeld: als u zeker bent dat u geen €1.000 per maand zou willen betalen voor dit medicijn, maar er misschien wel €750 per maand voor over zou hebben, kiest u €1.000).

| €0 | €10 | €15 | €25 | €50 | €75 | €100 | €125 | €150 | €200 | €250 | €300 | €500 | €750 | €1000 | €1500 | €2500 | … |
| --- | --- | --- | --- | --- | --- | --- | --- | --- | --- | --- | --- | --- | --- | --- | --- | --- | --- |
| O | O | O | O | O | O | O | O | O | O | O | O | O | O | O | O | O | O |

### B.1.C WTP changes length of life +25, 1 year

U heeft aangegeven dat u zeker 12 maanden [answer question B.1.A] per maand, maar zeker geen [answer question B.1.B] per maand wilt betalen voor een medicijn dat zorgt dat u één jaar langer leeft in kwaliteit van leven van 25 op de schaal van 0 (dood) tot 100 (perfecte gezondheid).

Wilt u het bedrag aangeven (tussen [answer question B.1.A] en [answer question B.1.B]) dat het maximale wat u bereid bent om te betalen voor dit medicijn het best benadert?

**< open question, answer should be a number within the range [answer question B.1.A] - [answer question B.1.B >**

### B.1.D WTP changes length of life +25, 1 year [only if B.1.C =0]

U heeft aangegeven dat u niet bereid bent meer dan €0 te betalen voor een medicijn dat ervoor zorgt dat u één jaar langer leeft in kwaliteit van leven van 25 op de schaal van 0 (dood) tot 100 (perfecte gezondheid). Wat is uw belangrijkste reden hiervoor?

- Ik kan niet meer dan €0 betalen
- Medicijnen zijn niet meer dan €0 waard voor mij
- Ik vind de medicijnen meer dan €0 waard, maar geef mijn geld liever uit aan iets anders
- Ik vind dat deze kosten vanuit de basis zorgverzekering zouden moeten worden vergoed
- Ik gebruik (liever) geen medicijnen
- De waarde van zorg en gezondheid is voor mij niet in geld uit te drukken
- Anders, namelijk **<open question >**

## B.2 WTP LL +50, 6 months

### B.2.A WTP changes length of life +50, 6 months

Stelt u zich voor dat u door ziekte nog één jaar te leven heeft in een kwaliteit van leven van 50 op de schaal van 0 (dood) tot 100 (volledig gezond). Een medicijn kan ervoor zorgen dat u zes maanden langer leeft in kwaliteit van leven van 50.

Hieronder ziet u een figuur die deze situatie en de levensverlenging door dit medicijn laat zien.

Voor deze levensverlenging moet u het medicijn (zonder bijwerkingen) een jaar lang elke maand innemen. U leeft dan zeker zes maanden langer in een kwaliteit van leven van 50. U moet het medicijn zelf betalen, uit uw eigen (gezins)inkomen. De betaling vindt plaats in 12 maandelijkse termijnen (u betaalt dus niet voor dit medicijn in de periode dat u langer leeft). Uw inkomen is in de gehele periode gelijk aan uw huidige inkomen.

Wilt u de onderstaande bedragen afgaan, van laag naar hoog, en het hoogste bedrag kiezen dat u zeker wel **per maand** zou willen betalen voor dit medicijn? (bijvoorbeeld: als u zeker bent dat u €100 per maand zou willen betalen voor dit medicijn, maar niet zeker of u er €150 per maand voor over zou hebben, kiest u €100).

| €0 | €10 | €15 | €25 | €50 | €75 | €100 | €125 | €150 | €200 | €250 | €300 | €500 | €750 | €1000 | €1500 | €2500 | … |
| --- | --- | --- | --- | --- | --- | --- | --- | --- | --- | --- | --- | --- | --- | --- | --- | --- | --- |
| O | O | O | O | O | O | O | O | O | O | O | O | O | O | O | O | O | O |

### B.2.B WTP changes length of life +50, 6 months

U heeft aangegeven dat u zeker 12 maanden [answer question B.2.A] per maand wilt betalen voor een medicijn dat zorgt dat u zes maanden langer leeft in kwaliteit van leven van 50 op de schaal van 0 (dood) tot 100 (perfecte gezondheid).

Wilt u de onderstaande bedragen afgaan, van laag naar hoog, en het laagste bedrag kiezen dat u zeker niet **per maand** zou willen betalen voor dit medicijn? (bijvoorbeeld: als u zeker bent dat u geen €1.000 per maand zou willen betalen voor dit medicijn, maar er misschien wel €750 per maand voor over zou hebben, kiest u €1.000).

| €0 | €10 | €15 | €25 | €50 | €75 | €100 | €125 | €150 | €200 | €250 | €300 | €500 | €750 | €1000 | €1500 | €2500 | … |
| --- | --- | --- | --- | --- | --- | --- | --- | --- | --- | --- | --- | --- | --- | --- | --- | --- | --- |
| O | O | O | O | O | O | O | O | O | O | O | O | O | O | O | O | O | O |

### B.2.C WTP changes length of life +50, 6 months

U heeft aangegeven dat u zeker 12 maanden [answer question B.2.A] per maand, maar zeker geen [answer question B.2.B] per maand wilt betalen voor een medicijn dat zorgt dat u zes maanden langer leeft in kwaliteit van leven van 50 op de schaal van 0 (dood) tot 100 (perfecte gezondheid).

Wilt u het bedrag aangeven (tussen [answer question B.2.A] en [answer question B.2.B]) dat het maximale wat u bereid bent om te betalen voor dit medicijn het best benadert?

**< open question, answer should be a number within the range [answer question B.2.A] - [answer question B.2.B >**

### B.2.D WTP changes length of life +50, 6 months [only if B.2.C =0]

U heeft aangegeven dat u niet bereid bent meer dan €0 te betalen voor een medicijn dat ervoor zorgt dat u zes maanden langer leeft in kwaliteit van leven van 50 op de schaal van 0 (dood) tot 100 (perfecte gezondheid). Wat is uw belangrijkste reden hiervoor?

- Ik kan niet meer dan €0 betalen
- Medicijnen zijn niet meer dan €0 waard voor mij
- Ik vind de medicijnen meer dan €0 waard, maar geef mijn geld liever uit aan iets anders
- Ik vind dat deze kosten vanuit de basis zorgverzekering zouden moeten worden vergoed
- Ik gebruik (liever) geen medicijnen
- De waarde van zorg en gezondheid is voor mij niet in geld uit te drukken
- Anders, namelijk **<open question >**

## B.3 WTP LL +75, 4 months

### B.3.A WTP changes length of life +75, 4 months

Stelt u zich voor dat u door ziekte nog één jaar te leven heeft in een kwaliteit van leven van 75 op de schaal van 0 (dood) tot 100 (volledig gezond). Een medicijn kan ervoor zorgen dat u vier maanden langer leeft in kwaliteit van leven van 75.

Hieronder ziet u een figuur die deze situatie en de levensverlenging door dit medicijn laat zien.

Voor deze levensverlenging moet u het medicijn (zonder bijwerkingen) een jaar lang elke maand innemen. U leeft dan zeker vier maanden langer in een kwaliteit van leven van 75. U moet het medicijn zelf betalen, uit uw eigen (gezins)inkomen. De betaling vindt plaats in 12 maandelijkse termijnen (u betaalt dus niet voor dit medicijn in de periode dat u langer leeft). Uw inkomen is in de gehele periode gelijk aan uw huidige inkomen.

Wilt u de onderstaande bedragen afgaan, van laag naar hoog, en het hoogste bedrag kiezen dat u zeker wel **per maand** zou willen betalen voor dit medicijn? (bijvoorbeeld: als u zeker bent dat u €100 per maand zou willen betalen voor dit medicijn, maar niet zeker of u er €150 per maand voor over zou hebben, kiest u €100).

| €0 | €10 | €15 | €25 | €50 | €75 | €100 | €125 | €150 | €200 | €250 | €300 | €500 | €750 | €1000 | €1500 | €2500 | … |
| --- | --- | --- | --- | --- | --- | --- | --- | --- | --- | --- | --- | --- | --- | --- | --- | --- | --- |
| O | O | O | O | O | O | O | O | O | O | O | O | O | O | O | O | O | O |

### B.3.B WTP changes length of life +75, 4 months

U heeft aangegeven dat u zeker 12 maanden [answer question B.3.A] per maand wilt betalen voor een medicijn dat zorgt dat u vier maanden langer leeft in kwaliteit van leven van 75 op de schaal van 0 (dood) tot 100 (perfecte gezondheid).

Wilt u de onderstaande bedragen afgaan, van laag naar hoog, en het laagste bedrag kiezen dat u zeker niet **per maand** zou willen betalen voor dit medicijn? (bijvoorbeeld: als u zeker bent dat u geen €1.000 per maand zou willen betalen voor dit medicijn, maar er misschien wel €750 per maand voor over zou hebben, kiest u €1.000).

| €0 | €10 | €15 | €25 | €50 | €75 | €100 | €125 | €150 | €200 | €250 | €300 | €500 | €750 | €1000 | €1500 | €2500 | … |
| --- | --- | --- | --- | --- | --- | --- | --- | --- | --- | --- | --- | --- | --- | --- | --- | --- | --- |
| O | O | O | O | O | O | O | O | O | O | O | O | O | O | O | O | O | O |

### B.3.C WTP changes length of life +75, 4 months

U heeft aangegeven dat u zeker 12 maanden [answer question B.3.A] per maand, maar zeker geen [answer question B.3.B] per maand wilt betalen voor een medicijn dat zorgt dat u vier maanden langer leeft in kwaliteit van leven van 75 op de schaal van 0 (dood) tot 100 (perfecte gezondheid).

Wilt u het bedrag aangeven (tussen [answer question B.3.A] en [answer question B.3.B]) dat het maximale wat u bereid bent om te betalen voor dit medicijn het best benadert?

**< open question, answer should be a number within the range [answer question B.3.A] - [answer question B.3.B >**

### B.3.D WTP changes length of life +75, 4 months [only if B.3.C =0]

U heeft aangegeven dat u niet bereid bent meer dan €0 te betalen voor een medicijn dat ervoor zorgt dat u vier maanden langer leeft in kwaliteit van leven van 75 op de schaal van 0 (dood) tot 100 (perfecte gezondheid). Wat is uw belangrijkste reden hiervoor?

- Ik kan niet meer dan €0 betalen
- Medicijnen zijn niet meer dan €0 waard voor mij
- Ik vind de medicijnen meer dan €0 waard, maar geef mijn geld liever uit aan iets anders
- Ik vind dat deze kosten vanuit de basis zorgverzekering zouden moeten worden vergoed
- Ik gebruik (liever) geen medicijnen
- De waarde van zorg en gezondheid is voor mij niet in geld uit te drukken
- Anders, namelijk **<open question >**

# C: Part 2 - Follow up questions WTP

Dit was het eerste deel van de vragenlijst. In het tweede deel van de vragenlijst stellen wij u enkele vragen over de zaken waar u rekening mee heeft gehouden in het eerste deel van de vragenlijst.

## C.1 Follow-up WTP [multiple answers possible]

U heeft in het eerste deel van de vragenlijst aangegeven wat u zou willen betalen voor verschillende medicijnen. Met welke zaken hield u hierbij rekening? U kunt meerdere antwoorden kiezen.

- Mijn productiviteit in de verschillende situaties (hoe goed ik in de verschillende situaties mijn werk kan doen)
- De tijd beschikbaar voor vrije tijd in de verschillende situaties
- Mijn bestedingspatroon in de verschillende situaties (de manier waarop ik in de verschillende situaties mijn geld besteed)
- Mijn nut van consumptie in de verschillende situaties (de tevredenheid die ik in de verschillende situaties ervaar bij het kopen en verbruiken van goederen en diensten*)
- Geen van bovenstaande opties

*Het kan bijvoorbeeld zo zijn dat u minder plezier hebt van zaken als eten, sport, hobby’s, uitgaan of van vakanties wanneer uw gezondheid slechter is en u daardoor minder tevredenheid ervaart. Tegelijkertijd zou u juist meer voldoening kunnen halen uit meer praktische diensten zoals huishoudelijke hulp waardoor uw tevredenheid toeneemt. Met nut van consumptie bedoelen wij steeds uw algehele tevredenheid met uw totale consumptie.

### C.1.B Follow-up WTP - productivity

#### **C.1.B.1 Follow-up WTP – productivity 1** [only if option 2 in C.1 is chosen]

U gaf aan dat u ook dacht aan uw **productiviteit** (hoe goed u uw werk zou kunnen doen) bij het beantwoorden van de vragen. Wat waren uw verwachtingen over de invloed van veranderingen in uw kwaliteit van leven op uw productiviteit? Kies het antwoord dat het beste past.

- Ik verwachtte dat mijn productiviteit *niet zou veranderen*
- Ik verwachtte dat een slechtere gezondheid mijn productiviteit *zou verlagen*
- Ik verwachtte dat een slechtere gezondheid mijn productiviteit *zou verhogen*
- Anders, namelijk <open question>

#### **C.1.B.2 Follow-up WTP – productivity 2** [only if option 2 in C.1 is chosen]

Wat waren uw verwachtingen over uw **productiviteit** in de verschillende situaties waar u langer zou leven (het gaat hier dus over uw productiviteit in de periode dat u extra zou leven)? Kies het antwoord dat het beste past.

- Ik verwachtte dat mijn productiviteit *gelijk* zou zijn aan mijn productiviteit in het jaar daarvoor
- Ik verwachtte dat mijn productiviteit *lager* zou zijn dan mijn productiviteit in het jaar daarvoor
- Ik verwachtte dat mijn productiviteit *hoger* zou zijn dan mijn productiviteit in het jaar daarvoor
- Anders, namelijk <open question>

#### **C.1.B.3 Follow-up WTP – productivity 3** [only if option 2 in C.1 is chosen]

In hoeverre heeft u uw **productiviteit** in de verschillende situaties laten meewegen in uw antwoorden? Kies het antwoord dat het beste past.

- Productiviteit heeft mijn antwoorden *niet of nauwelijks* beïnvloed
- Productiviteit heeft mijn antwoorden *een beetje* beïnvloed
- Productiviteit heeft mijn antwoorden *sterk* beïnvloed

#### **C.1.B.4 Follow-up WTP – productivity 4** [only if option 2 in C.1 is NOT chosen]

U gaf aan dat u niet dacht aan uw **productiviteit** bij het beantwoorden van de vragen. Wat is hiervoor uw reden? Kies het antwoord dat het beste past.

- Dit werd niet specifiek gevraagd
- Er was geen informatie beschikbaar over productiviteit
- Ik heb hier niet over nagedacht
- Anders, namelijk **<open question>**

### C.1.C Follow-up WTP - leisure

#### **C.1.C.1 Follow-up WTP – leisure 1** [only if option 3 in C.1 is chosen]

U gaf aan dat u ook dacht aan de **tijd beschikbaar voor vrije tijd** bij het beantwoorden van de vragen. Wat waren uw verwachtingen over de invloed van veranderingen in uw kwaliteit van leven op uw tijd beschikbaar voor vrije tijd? Kies het antwoord dat het beste past.

- Ik verwachtte dat de tijd beschikbaar voor vrije tijd *niet zou veranderen*
- Ik verwachtte dat een slechtere gezondheid zou zorgen voor *minder* tijd beschikbaar voor vrije tijd
- Ik verwachtte dat een slechtere gezondheid zou zorgen voor *meer* tijd beschikbaar voor vrije tijd
- Anders, namelijk <open question>

#### **C.1.C.2 Follow-up WTP – leisure 2** [only if option 3 in C.1 is chosen]

Wat waren uw verwachtingen over de **tijd beschikbaar voor vrije tijd** in de verschillende situaties waar u langer zou leven (het gaat hier dus over de tijd beschikbaar voor vrije tijd in de periode dat u extra zou leven)? Kies het antwoord dat het beste past.

- Ik verwachtte dat ik *evenveel* tijd beschikbaar zou hebben voor vrije tijd als in het jaar daarvoor
- Ik verwachtte dat ik *minder* tijd beschikbaar zou hebben voor vrije tijd dan in het jaar daarvoor
- Ik verwachtte dat ik *meer* tijd beschikbaar zou hebben voor vrije tijd dan in het jaar daarvoor
- Anders, namelijk <open question>

#### **C.1.C.3 Follow-up WTP – leisure 3** [only if option 3 in C.1 is chosen]

In hoeverre heeft u de **tijd beschikbaar voor vrije tijd** in de verschillende situaties laten meewegen in uw antwoorden? Kies het antwoord dat het beste past.

- De tijd beschikbaar voor vrije tijd heeft mijn antwoorden *niet of nauwelijks* beïnvloed
- De tijd beschikbaar voor vrije tijd heeft mijn antwoorden *een beetje* beïnvloed
- De tijd beschikbaar voor vrije tijd heeft mijn antwoorden *sterk* beïnvloed

#### **C.1.C.4 Follow-up WTP – leisure 4** [only if option 3 in C.1 is NOT chosen]

U gaf aan dat u niet dacht aan de **tijd beschikbaar voor vrije tijd** bij het beantwoorden van de vragen. Wat is hiervoor uw reden? Kies het antwoord dat het beste past.

- Dit werd niet specifiek gevraagd
- Er was geen informatie beschikbaar over de tijd beschikbaar voor vrije tijd
- Ik heb hier niet over nagedacht
- Anders, namelijk **<open question>**

### **C.1.D Follow-up WTP - consumption**

#### **C.1.D.1 Follow-up WTP – consumption 1** [only if option 4 in C.1 is chosen]

U gaf aan dat u ook dacht aan uw **bestedingspatroon** (de manier waarop u uw geld zou besteden) bij het beantwoorden van de vragen. Wat waren uw verwachtingen over de invloed van veranderingen in uw kwaliteit van leven op uw bestedingspatroon? Kies het antwoord dat het beste past.

- Ik verwachtte dat mijn bestedingspatroon *niet zou veranderen*
- Ik verwachtte dat ik in een slechtere gezondheid in totaal *minder* zou besteden
- Ik verwachtte dat ik in een slechtere gezondheid in totaal *meer* zou besteden
- Ik verwachtte dat ik in totaal *evenveel* zou besteden in een slechtere gezondheid, *maar dat ik mijn geld wel aan andere dingen zou uitgeven*
- Anders, namelijk **<open question>**

#### **C.1.D.2 Follow-up WTP – consumption 2** [only if option 4 in C.1 is chosen AND option 2, 3, 4, or 5 in C.1.D.1]

Op welke van de volgende categorieën bestedingen dacht u dat veranderingen in uw kwaliteit van leven invloed zouden hebben? U kunt meerdere antwoorden kiezen.

- Voeding
- Dranken en rookwaren
- Kleding en schoeisel
- Wonen
- Woninginrichting en huisraad
- Gezondheidszorg
- Vervoer en communicatie
- Recreatie en ontwikkeling
- Overig

#### **C.1.D.3 Follow-up WTP – consumption 3** [only if option 4 in C.1 is chosen AND option 2, 3, 4, or 5 in C.1.D.1]

U gaf aan dat de u dacht dat de veranderingen in uw kwaliteit van leven invloed zouden hebben op de categorie(ën) hieronder. Zou u meer of minder uitgeven aan deze categorie(ën)?

**Meer** **Minder**

- Voeding □ □
- Dranken en rookwaren □ □
- Kleding en schoeisel □ □
- Wonen □ □
- Woninginrichting en huisraad □ □
- Gezondheidszorg □ □
- Vervoer en communicatie □ □
- Recreatie en ontwikkeling □ □
- Overig □ □

#### **C.1.D.4 Follow-up WTP – consumption 4** [only if option 4 in C.1 is chosen]

Wat waren uw verwachtingen over uw **bestedingspatroon** in de verschillende situaties waar u langer zou leven (het gaat hier dus over uw bestedingspatroon in de periode dat u extra zou leven)? Kies het antwoord dat het beste past.

- Ik verwachtte dat mijn bestedingspatroon *gelijk* zou zijn aan dat in het jaar daarvoor
- Ik verwachtte dat ik in totaal *minder* zou besteden dan in het jaar daarvoor
- Ik verwachtte dat ik in totaal *meer* zou besteden dan in het jaar daarvoor
- Ik verwachtte dat ik in totaal *evenveel* zou besteden als in het jaar daarvoor, *maar dat ik mijn geld wel aan andere dingen zou uitgeven*
- Anders, namelijk <open question>

#### **C.1.D.5 Follow-up WTP – consumption 5** [only if option 4 in C.1 is chosen AND option 2, 3, 4, or 5 in C.1.D.4]

Op welke van de volgende categorieën bestedingen dacht u dat de verschillende situaties (waar u langer zou leven) invloed hadden? U kunt meerdere antwoorden kiezen.

- Voeding
- Dranken en rookwaren
- Kleding en schoeisel
- Wonen
- Woninginrichting en huisraad
- Gezondheidszorg
- Vervoer en communicatie
- Recreatie en ontwikkeling
- Overig

#### **C.1.D.6 Follow-up WTP – consumption 6** [only if option 4 in C.1 is chosen AND option 2, 3, 4, or 5 in C.1.D.4]

U gaf aan dat de situaties (waar u langer zou leven) uw bestedingen in de categorie(ën) hieronder zou beïnvloeden. Zou u meer of minder uitgeven aan deze categorie(ën)?

**Meer** **Minder**

- Voeding □ □
- Dranken en rookwaren □ □
- Kleding en schoeisel □ □
- Wonen □ □
- Woninginrichting en huisraad □ □
- Gezondheidszorg □ □
- Vervoer en communicatie □ □
- Recreatie en ontwikkeling □ □
- Overig □ □

#### **C.1.D.7 Follow-up WTP – consumption 7** [only if option 4 in C.1 is chosen]

In hoeverre heeft u uw **bestedingspatroon** in de verschillende situaties laten meewegen in uw antwoorden? Kies het antwoord dat het beste past.

- Mijn bestedingspatroon heeft mijn antwoorden *niet of nauwelijks* beïnvloed
- Mijn bestedingspatroon heeft mijn antwoorden *een beetje* beïnvloed
- Mijn bestedingspatroon heeft mijn antwoorden *sterk* beïnvloed

#### **C.1.D.8 Follow-up WTP – consumption 8** [only if option 4 in C.1 is NOT chosen]

U gaf aan dat u niet dacht aan uw **bestedingspatroon** bij het beantwoorden van de vragen. Wat is hiervoor uw reden? Kies het antwoord dat het beste past.

- Dit werd niet specifiek gevraagd
- Er was geen informatie beschikbaar over bestedingspatronen
- Ik heb hier niet over nagedacht
- Anders, namelijk **<open question>**

### C.1.E Follow-up WTP – utility consumption

#### **C.1.E.1 Follow-up WTP – utility consumption 1** [only if option 5 in C.1 is chosen]

U gaf aan dat u ook dacht aan uw **nut van consumptie** bij het beantwoorden van de vragen. Wat waren uw verwachtingen over de invloed van veranderingen in uw kwaliteit van leven op uw nut van consumptie? Kies het antwoord dat het beste past.

- Ik verwachtte dat mijn nut van consumptie *niet zou veranderen*
- Ik verwachtte dat een slechtere gezondheid mijn nut van consumptie zou *verlagen*
- Ik verwachtte dat een slechtere gezondheid mijn nut van consumptie zou *verhogen*
- Anders, namelijk <open question>

#### **C.1.E.2 Follow-up WTP – utility consumption 1** [only if option 5 in C.1 is chosen]

Wat waren uw verwachtingen over uw **nut van consumptie** in de verschillende situaties waar u langer zou leven (het gaat hier dus over uw nut van consumptie in de periode dat u extra zou leven)? Kies het antwoord dat het beste past.

- Ik verwachtte dat mijn nut van consumptie *gelijk* zou zijn aan mijn nut van consumptie in het jaar daarvoor
- Ik verwachtte dat mijn nut van consumptie *lager* zou zijn dan mijn nut van consumptie in het jaar daarvoor
- Ik verwachtte dat mijn nut van consumptie *hoger* zou zijn dan mijn nut van consumptie in het jaar daarvoor
- Anders, namelijk <open question>

#### **C.1.E.3 Follow-up WTP – utility consumption 2** [only if option 5 in C.1 is chosen]

In hoeverre heeft u uw **nut van consumptie** in de verschillende situaties laten meewegen bij het bepalen van uw bereidheid om te betalen voor de medicijnen? Kies het antwoord dat het beste past.

- Nut van consumptie heeft mijn antwoorden *niet of nauwelijks* beïnvloed
- Nut van consumptie heeft mijn antwoorden *een beetje* beïnvloed
- Nut van consumptie heeft mijn antwoorden *sterk* beïnvloed

#### **C.1.E.4 Follow-up WTP – utility consumption** **3** [only if option 5 in C.1 is NOT chosen]

U heeft aangegeven dat u uw nut van consumptie in de verschillende situaties niet heeft meegenomen bij het bepalen van uw bereidheid om te betalen voor de medicijnen. Wat is hiervoor uw reden? Kies het antwoord dat het beste past.

- Dit werd niet specifiek gevraagd
- Er was geen informatie beschikbaar over het nut van consumptie
- Ik heb hier niet over nagedacht

Anders, namelijk <open question>

# D: Part 3 - Introduction and questions utility of consumption scale

Dit was het tweede deel van de vragenlijst. In het derde deel van de vragenlijst stellen wij u enkele vragen over uw nut van consumptie in de verschillende gezondheidstoestanden. Eerst volgen enkele voorbeelden om de nut van consumptie schaal die in de vragen wordt gebruikt uit te leggen.

## D.1 Introduction utility of consumption scale

### D.1.A Introduction utility of consumption scale I

Uw nut van consumptie kan verschillen in verschillende gezondheidstoestanden. Met nut van consumptie bedoelen wij de tevredenheid die u ervaart bij het kopen en verbruiken van goederen en diensten.

Het kan bijvoorbeeld zo zijn dat u minder plezier hebt van zaken als eten, sport, hobby’s, uitgaan of van vakanties wanneer uw gezondheid slechter is en u daardoor minder tevredenheid ervaart. Tegelijkertijd zou u juist meer voldoening kunnen halen uit meer praktische diensten zoals huishoudelijke hulp waardoor uw tevredenheid toeneemt. Met nut van consumptie bedoelen wij steeds uw algehele tevredenheid met uw totale consumptie.

Om nut van consumptie uit te drukken gebruiken wij een nut van consumptie schaal. Hieronder ziet u een figuur die de schaal voor nut van consumptie laat zien. U kunt dit zien als een nutsthermometer met onderaan ‘geen nut’ (score 0) en bovenaan ‘hoogst haalbare nut’ (score 100).

### D.1.B Introduction utility of consumption scale II (own utility of consumption)

Hoe zou u uw huidige nut van consumptie beoordelen op de schaal van 0 (geen nut) tot 100 (hoogst haalbare nut))?

Geef op de schaal in de figuur hieronder aan welk getal uw nut het beste weergeeft. Dit doet u door op het bolletje te klikken en dit met uw muis ingedrukt naar boven of beneden te schuiven.

**< answer should be in range 0-100, answer should become visible on scale in figure below>**

### D.1.C Introduction utility of consumption scale III

In de vragen hierna wordt ook de lengte van leven aangegeven. Hieronder ziet u een figuur die één levensjaar met een nut van consumptie van 100 op de schaal van 0 (geen nut) tot 100 (hoogst haalbare nut)) laat zien.

### D.1.D Introduction utility of consumption scale IV

Stelt u zich voor dat u door een ziekte nog één jaar te leven heeft en dat uw nut van consumptie in dit jaar 50 is op de schaal van 0 (geen nut) tot 100 (hoogst haalbare nut)). Hieronder ziet u een figuur die deze situatie laat zien.

## D.2 Questions utility of consumption

Dit was het laatste voorbeeld voor het derde deel van de vragenlijst. Wij vragen u hierna aan te geven hoe hoog u denkt dat uw nut van consumptie zou zijn in verschillende gezondheidstoestanden.

### D.2.A Utility of consumption for quality of life 25

Stelt u zich voor dat u door ziekte het komende jaar leeft in een kwaliteit van leven van 25 op de schaal van 0 (dood) tot 100 (volledig gezond). Deze situatie is uitgebeeld in de figuur links hieronder.

Hoe hoog denkt u dat uw nut van consumptie zou zijn in dit jaar in deze gezondheidstoestand op de schaal van 0 (geen nut) tot 100 (hoogst haalbare nut))? Ga hierbij uit van uw huidige inkomen.

Geef op de schaal in de figuur rechts hieronder aan welk getal uw nut het beste weergeeft. Dit doet u door op het antwoordblokje te klikken en dit met uw muis ingedrukt naar boven of beneden te schuiven.

**<Open question, answer should be in range 0-100, answer should be visible in figure right below in a manner equal to figure left below (in that figure, answer would be 25)>**

### D.2.B Utility of consumption for quality of life 50

Stelt u zich voor dat u door ziekte het komende jaar leeft in een kwaliteit van leven van 50 op de schaal van 0 (dood) tot 100 (volledig gezond). Deze situatie is uitgebeeld in de figuur links hieronder.

Hoe hoog denkt u dat uw nut van consumptie is in dit jaar in deze gezondheidstoestand op de schaal van 0 (geen nut) tot 100 (hoogst haalbare nut))? Ga hierbij uit van uw huidige inkomen.

Geef op de schaal in de figuur rechts hieronder aan welk getal uw nut het beste weergeeft. Dit doet u door op het antwoordblokje te klikken en dit met uw muis ingedrukt naar boven of beneden te schuiven.

**<Open question, answer should be in range 0-100, answer should be visible in figure right below in a manner equal to figure left below (in that figure, answer would be 50)>**

### D.2.C Utility of consumption for quality of life 75

Stelt u zich voor dat u door ziekte het komende jaar leeft in een kwaliteit van leven van 75 op de schaal van 0 (dood) tot 100 (volledig gezond). Deze situatie is uitgebeeld in de figuur links hieronder.

Hoe hoog denkt u dat uw nut van consumptie is in dit jaar in deze gezondheidstoestand op de schaal van 0 (geen nut) tot 100 (hoogst haalbare nut))? Ga hierbij uit van uw huidige inkomen.

Geef op de schaal in de figuur rechts hieronder aan welk getal uw nut het beste weergeeft. Dit doet u door op het antwoordblokje te klikken en dit met uw muis ingedrukt naar boven of beneden te schuiven.

**<Open question, answer should be in range 0-100, answer should be visible in figure right below in a manner equal to figure left below (in that figure, answer would be 75)>**

### D.2.D Utility of consumption for quality of life 100

Stelt u zich voor dat u het komende jaar leeft in een kwaliteit van leven van 100 op de schaal van 0 (dood) tot 100 (volledig gezond). Deze situatie is uitgebeeld in de figuur links hieronder.

Hoe hoog denkt u dat uw nut van consumptie is in dit jaar in deze gezondheidstoestand op de schaal die loopt van 0 (geen nut) tot 100 (hoogst haalbare nut))? Ga hierbij uit van uw huidige inkomen.

Geef op de schaal in de figuur rechts hieronder aan welk getal uw nut het beste weergeeft. Dit doet u door op het antwoordblokje te klikken en dit met uw muis ingedrukt naar boven of beneden te schuiven.

**<Open question, answer should be in range 0-100, answer should be visible in figure right below in a manner equal to figure left below (in that figure, answer would be 100)>**

## D.3 Questions utility of consumption + money gain

In de volgende vragen beschrijven wij steeds een gezondheidstoestand met het bijbehorende nut van consumptie uit uw vorige antwoorden. Vervolgens vragen wij u hoe u denkt dat uw nut van consumptie zou veranderen wanneer u een geldbedrag zou winnen.

### D.3.A Utility of consumption for quality of life 25 + money gain

Stelt u zich voor dat u door ziekte het komende jaar leeft in een kwaliteit van leven van 25 op de schaal van 0 (dood) tot 100 (volledig gezond). Uw nut van consumptie in dit jaar zou [answer question D.2.A] zijn op de schaal van 0 (geen nut) tot 100 (hoogst haalbare nut)). Deze situatie is uitgebeeld in de figuur hieronder.

Stelt u zich nu voor dat u een geldbedrag wint ter waarde van €100.000. Hoe hoog denkt u dat uw nut van consumptie zou zijn na het winnen van dit geldbedrag? Ga naast dit bedrag uit van uw huidige inkomen.

Geef op de schaal in de figuur rechts hieronder aan welk getal uw nut het beste weergeeft. Dit doet u door op het antwoordblokje te klikken en dit met uw muis ingedrukt naar boven of beneden te schuiven.

**<Open question, answer should be in range 0-100, answer should be visible in the right figure below, see comment for specifics>**

### D.3.B Utility of consumption for quality of life 50 + money gain

Stelt u zich voor dat u door ziekte het komende jaar leeft in een kwaliteit van leven van 50 op de schaal van 0 (dood) tot 100 (volledig gezond). Uw nut van consumptie in dit jaar zou [answer question D.2.B] zijn op de schaal van 0 (geen nut) tot 100 (hoogst haalbare nut)). Deze situatie is uitgebeeld in de figuur hieronder.

Stelt u zich nu voor dat u een geldbedrag wint ter waarde van €100.000. Hoe hoog denkt u dat uw nut van consumptie zou zijn na het winnen van dit geldbedrag? Ga naast dit bedrag uit van uw huidige inkomen.

Geef op de schaal in de figuur rechts hieronder aan welk getal uw nut het beste weergeeft. Dit doet u door op het antwoordblokje te klikken en dit met uw muis ingedrukt naar boven of beneden te schuiven.

**<Open question, answer should be in range 0-100, answer should be visible in the right figure below, see comment for D.3.A for an example>**

### D.3.C Utility of consumption for quality of life 75 + money gain

Stelt u zich voor dat u door ziekte het komende jaar leeft in een kwaliteit van leven van 75 op de schaal van 0 (dood) tot 100 (volledig gezond). Uw nut van consumptie in dit jaar zou [answer question D.2.C] zijn op de schaal van 0 (geen nut) tot 100 (hoogst haalbare nut)). Deze situatie is uitgebeeld in de figuur hieronder.

Stelt u zich nu voor dat u een geldbedrag wint ter waarde van €100.000. Hoe hoog denkt u dat uw nut van consumptie zou zijn na het winnen van dit geldbedrag? Ga naast dit bedrag uit van uw huidige inkomen.

Geef op de schaal in de figuur rechts hieronder aan welk getal uw nut het beste weergeeft. Dit doet u door op het antwoordblokje te klikken en dit met uw muis ingedrukt naar boven of beneden te schuiven.

**<Open question, answer should be in range 0-100, answer should be visible in the right figure below, see comment for D.3.A for an example>**

### D.3.D Utility of consumption for quality of life 100 + money gain

Stelt u zich voor dat u het komende jaar leeft in een kwaliteit van leven van 100 op de schaal van 0 (dood) tot 100 (volledig gezond). Uw nut van consumptie in dit jaar zou [answer question D.2.D] zijn op de schaal van 0 (geen nut) tot 100 (hoogst haalbare nut)). Deze situatie is uitgebeeld in de figuur hieronder.

Stelt u zich nu voor dat u een geldbedrag wint ter waarde van €100.000. Hoe hoog denkt u dat uw nut van consumptie zou zijn na het winnen van dit geldbedrag? Ga naast dit bedrag uit van uw huidige inkomen.

Geef op de schaal in de figuur rechts hieronder aan welk getal uw nut het beste weergeeft. Dit doet u door op het antwoordblokje te klikken en dit met uw muis ingedrukt naar boven of beneden te schuiven.

**<Open question, answer should be in range 0-100, answer should be visible in the right figure below, see comment for D.3.A for an example>**

## D.4 Questions income for changing health

### D.4.A Expectations income for changing health QoL

Wij hebben u hiervoor steeds gevraagd om uit te gaan van uw huidige inkomen. Hoe denkt u dat uw inkomen in het komende jaar zou veranderen als uw kwaliteit van leven zou veranderen? Kies het antwoord dat het beste past.

- Ik verwacht dat mijn inkomen *niet zou veranderen*
- Ik verwacht dat een slechtere gezondheid mijn inkomen *zou verlagen*
- Ik verwacht dat een slechtere gezondheid mijn inkomen *zou verhogen*
- Anders, namelijk <open question>

### D.4.B Expectations income for changing health Length

Wij hebben u ook gevraagd om uit te gaan van uw huidige inkomen wanneer wij u vroegen u voor te stellen dat u door een medicijn langer zou leven. Wat denkt u dat uw inkomen zou zijn in de periode dat u langer zou leven? Kies het antwoord dat het beste past.

- Ik verwacht dat mijn inkomen *gelijk* zou zijn aan mijn inkomen in het jaar daarvoor
- Ik verwacht dat mijn inkomen *lager* zou zijn dan mijn inkomen in het jaar daarvoor
- Ik verwacht dat mijn inkomen *hoger* zou zijn dan mijn inkomen in het jaar daarvoor
- Anders, namelijk <open question>

# E: Part 4 - Respondent information

Dit was het derde deel van de vragenlijst. In het vierde en laatste deel stellen wij u enkele vragen over uzelf.

## E.1 Age

Wat is uw leeftijd?

**<open question, answer must be a number>**

## E.2 Gender

Wat is uw geslacht?

- Man
- Vrouw

## E.3 Highest education

Wat is uw hoogst afgeronde opleiding?

- Geen opleiding
- Lagere school/ Basisonderwijs
- Lager of voorbereidend beroepsonderwijs: LBO, VBO, LTS. LHNO, VMBO
- Middelbaar algemeen voortgezet onderwijs: MAVO, VMBO-t, MBO-kort
- Middelbaar beroepsonderwijs: MBO, MTS, MEAO
- Hoger algemeen voortgezet onderwijs: HAVO, VWO, Gymnasium
- Hoger beroepsonderwijs: HBO, HEAO, HTS
- Wetenschappelijk onderwijs: Universiteit
- Anders, namelijk **<open question>**

## E.4 Marital status

Wat is uw huwelijkse staat?

- Alleenstaand, nooit getrouwd
- Samenwonend met partner
- Getrouwd, geregistreerd partnerschap
- Gescheiden
- Weduwe, weduwnaar

## E.5 Household size

Uit hoeveel personen bestaat uw huishouden? Reken uzelf mee.

**<open question, answer must be a number, minimum 1>**

## E.6 Children

Heeft u kinderen?

- Ja, ik heb **<open question, answer must be a number, minimum of 1>** kind(eren)
- Nee

## E.7 Children at home [only if E.6 = ‘Ja…’]

Hoeveel kinderen onder de 18 jaar wonen (deeltijd) bij u thuis?

❑ **<open question, answer must be a number>** kind(eren)

## E.8 Employment

Kies hieronder wat voor u tijdens een werkweek het meest van toepassing is. Waaraan besteedt u het meeste van uw tijd tijdens een werkweek?

- Ik ben in loondienst
- Ik ben zelfstandig ondernemer
- Ik ben werkloos, werkzoekend
- Ik ben met de VUT, (pre)pensioen of rentenierend
- Ik ben studerend, schoolgaand
- Ik ben huisman, huisvrouw
- Ik doe vrijwilligerswerk
- Anders, namelijk **<open question >**

## E.9 Net household income

Wat is het netto-inkomen per maand van uw huishouden?

**<open question, answer must be a number>**

## E.10 Income sufficient

Als u denkt aan het totale inkomen per maand van uw huishouden, hoe goed kan uw huishouden rondkomen?

- Met veel moeite
- Met enige moeite
- Vrij gemakkelijk
- Gemakkelijk

## E.11 Religion 1

Wat is uw geloofsovertuiging?

- Ik ben niet gelovig/atheïst/agnost
- Protestantschristelijk
- Katholiek christelijk
- Islamitisch
- Boeddhistisch
- Hindoeïstisch
- Anders, namelijk **<open question>**

## E.12 Religion 2

Hoe belangrijk is geloof voor u in uw dagelijkse leven?

- Volledig onbelangrijk
- Onbelangrijk
- Neutraal
- Belangrijk
- Zeer belangrijk

# F: End questionnaire

Dit is het einde van de vragenlijst. Bedankt voor uw deelname!
