# Supplementary material for: Benefits beyond health in the willingness to pay for a quality-adjusted life-year
Source: Eur J Health Econ. 2024 Oct 7;26(4):653–70. doi: 10.1007/s10198-024-01726-7 (PMC12126320; doi:10.1007/s10198-024-01726-7)
Supplement: Supplementary file 7 — Supplementary file7 (DOCX 238 kb) [file 10198_2024_1726_MOESM7_ESM.docx]

Version 4 – Instruction

# A: Introduction and permission

## A.1 Introduction to the questionnaire and permission

Het budget voor gezondheidszorg is beperkt. Beleidsmakers moeten daarom keuzes maken over welke behandelingen vergoed kunnen worden via de basis zorgverzekering. Bij het maken van deze keuzes houden beleidsmakers rekening met de waarde die Nederlanders hechten aan zorg en gezondheid. Dit onderzoek gaat hierover.

Het onderzoek wordt uitgevoerd door de Erasmus Universiteit Rotterdam. In deze vragenlijst wordt u gevraagd op verschillende manieren bepaalde gezondheidstoestanden en veranderingen te beoordelen. Probeer u bij het beantwoorden van deze vragen zo goed mogelijk voor te stellen wat deze gezondheidstoestanden en veranderingen voor u zouden betekenen. Er zijn geen goede of foute antwoorden, we zijn geïnteresseerd in uw mening.

Deze vragenlijst bestaat uit drie delen en deelname duurt ongeveer 20 minuten. Het is belangrijk dat u alle vragen zorgvuldig leest en op iedere vraag antwoord geeft. Uw antwoorden worden volledig anoniem verwerkt.

*Begrijpt u het doel van dit onderzoek, wilt u aan dit onderzoek deelnemen en geeft u ons toestemming om uw antwoorden anoniem te gebruiken voor wetenschappelijk onderzoek?*

- Ja
- Nee

*U kunt uw deelname hierna op ieder gewenst moment alsnog beëindigen.*

## A.2 Permission received [text only if A.1 = ‘Ja’]

Fijn dat u deze vragenlijst wilt invullen. Alvast bedankt voor uw medewerking. De vragenlijst start in het volgende scherm.

## A.3 Permission not received [text only if A.1 = ‘Nee’ 🡪 for these people this is the last screen]

Jammer dat u deze vragenlijst niet wilt invullen. Dit is voor u het einde van dit onderzoek.

# B: Part 1 - Introduction scale(s) + tasks

Als voorbereiding op het eerste deel van de vragenlijst geven wij hierna enkele voorbeelden van de scenario’s die door de vragenlijst heen worden gebruikt. Te beginnen met de omschrijving van de gezondheidstoestanden.

## B.1 Introduction health utility scale

### B.1.A Introduction health utility scale I

In de vragenlijst worden verschillende gezondheidstoestanden beschreven. De *kwaliteit van leven* in de gezondheidstoestanden wordt uitgedrukt in een score tussen 0 en 100. De score 0 staat gelijk aan de dood. De score 100 staat gelijk aan de kwaliteit van leven in perfecte gezondheid.

Het kan bijvoorbeeld zo zijn dat u door een ziekte veel pijn heeft en u ernstig wordt belemmerd in uw dagelijks leven en zelfstandigheid, waardoor uw kwaliteit van leven relatief laag is. Een ander voorbeeld is dat u af en toe last heeft van hoofdpijn, maar dit over het algemeen geen grote impact heeft op uw leven. Dit zou passen bij een relatief hoge kwaliteit van leven.

Hieronder ziet u een figuur die de kwaliteit van leven schaal laat zien. U kunt dit zien als een gezondheidsthermometer met onderaan ‘dood’ (score 0) en bovenaan ‘volledig gezond’ (score 100).

### B.1.B Introduction health utility scale II (own health utility)

Hoe zou u uw huidige gezondheid beoordelen op de schaal van 0 (dood) tot 100 (perfecte gezondheid)?

Geef op de schaal in de figuur hieronder aan welk getal uw gezondheid het beste weergeeft. Dit doet u door op het bolletje te klikken en dit met uw muis ingedrukt naar boven of beneden te schuiven.

**<Open question, answer should be in range 0-100, answer should become visible on scale in figure below>**

### B.1.C Introduction health utility scale III

Naast *kwaliteit van leven* wordt in de vragenlijst ook de *lengte van leven* aangegeven. Stelt u zich voor dat u nog *één jaar* te leven heeft in een kwaliteit van leven van 100 op de schaal van 0 (dood) tot 100 (volledig gezond). Hieronder ziet u een figuur die deze situatie laat zien.

### B.1.D Introduction health utility scale IV

Stelt u zich voor dat u door een ziekte nog één jaar te leven heeft in een kwaliteit van leven van 80 op de schaal van 0 (dood) tot 100 (volledig gezond). Hieronder ziet u een figuur die deze situatie laat zien.

### B.1.E Introduction health utility scale V

In de vragenlijst worden verschillende veranderingen in gezondheidstoestanden beschreven als gevolg van het gebruik van een medicijn. In dit voorbeeld beschrijven wij een verandering in de *kwaliteit van leven* door een medicijn.

Stelt u zich voor dat u door een ziekte nog één jaar te leven heeft in een kwaliteit van leven van 80 op de schaal van 0 (dood) tot 100 (volledig gezond). Een medicijn kan ervoor zorgen dat uw kwaliteit van leven in dit jaar stijgt van 80 naar 100.

Hieronder ziet u een figuur die deze situatie en de stijging van de kwaliteit van leven door dit medicijn laat zien.

### B.1.F Introduction health utility scale VI

In dit voorbeeld beschrijven wij een verandering in de *lengte van leven* als gevolg van het gebruik van een medicijn.

Stelt u zich voor dat u door ziekte nog één jaar te leven heeft in een kwaliteit van leven van 80 op de schaal van 0 (dood) tot 100 (volledig gezond). Een medicijn kan ervoor zorgen dat u één jaar langer leeft in kwaliteit van leven van 80.

Hieronder ziet u een figuur die deze situatie en de levensverlenging door dit medicijn laat zien.

## B.2 Introduction utility of consumption scale

### B.2.A Introduction utility of consumption scale I

Uw nut van consumptie kan verschillen in verschillende gezondheidstoestanden. Met nut van consumptie bedoelen wij de tevredenheid die u ervaart bij het kopen en verbruiken van goederen en diensten.

Het kan bijvoorbeeld zo zijn dat u minder plezier hebt van zaken als eten, sport, hobby’s, uitgaan of van vakanties wanneer uw gezondheid slechter is en u daardoor minder tevredenheid ervaart. Tegelijkertijd zou u juist meer voldoening kunnen halen uit meer praktische diensten zoals huishoudelijke hulp waardoor uw tevredenheid toeneemt. Met nut van consumptie bedoelen wij steeds uw algehele tevredenheid met uw totale consumptie.

Om nut van consumptie uit te drukken gebruiken wij een nut van consumptie schaal. Hieronder ziet u een figuur die de schaal voor nut van consumptie laat zien. U kunt dit zien als een nutsthermometer met onderaan ‘geen nut’ (score 0) en bovenaan ‘hoogst haalbare nut’ (score 100).

### B.2.B Introduction utility of consumption scale II (own utility of consumption)

Hoe zou u uw huidige nut van consumptie beoordelen op de schaal van 0 (geen nut) tot 100 (hoogst haalbare nut))?

Geef op de schaal in de figuur hieronder aan welk getal uw nut het beste weergeeft. Dit doet u door op het bolletje te klikken en dit met uw muis ingedrukt naar boven of beneden te schuiven.

**<Open question, answer should be in range 0-100, answer should become visible on scale in figure below>**

### B.2.C Introduction utility of consumption scale III

In de vragen wordt ook de lengte van leven aangegeven. Hieronder ziet u een figuur die één levensjaar met een nut van consumptie van 100 op de schaal van 0 (geen nut) tot 100 (hoogst haalbare nut)) laat zien.

### B.2.D Introduction utility of consumption scale IV

Stelt u zich voor dat u door een ziekte nog één jaar te leven heeft en dat uw nut van consumptie in dit jaar 50 is op de schaal van 0 (geen nut) tot 100 (hoogst haalbare nut)). Hieronder ziet u een figuur die deze situatie laat zien.

### B.2.E Introduction utility of consumption scale V

In de vragenlijst worden verschillende veranderingen in gezondheidstoestanden beschreven als gevolg van het gebruik van een medicijn. In dit voorbeeld beschrijven wij de invloed van een verandering van *kwaliteit van leven* op uw nut van consumptie.

Stelt u zich voor dat u door een ziekte nog één jaar te leven heeft en dat uw nut van consumptie in dit jaar 50 is op de schaal van 0 (geen nut) tot 100 (hoogst haalbare nut)). Een medicijn kan ervoor zorgen dat uw kwaliteit van leven stijgt en daarmee ook uw nut van consumptie. Uw nut van consumptie in de nieuwe situatie is 70.

Hieronder ziet u een figuur die deze situatie en de stijging van uw nut van consumptie door de stijging van de kwaliteit van leven door dit medicijn laat zien.

### B.2.F Introduction utility of consumption scale VI

In dit voorbeeld beschrijven wij de invloed van een verandering van *lengte van leven* op uw nut van consumptie

Stelt u zich voor dat u door een ziekte nog één jaar te leven heeft en dat uw nut van consumptie in dit jaar 50 is op de schaal van 0 (geen nut) tot 100 (hoogst haalbare nut)). Een medicijn kan ervoor zorgen dat u één jaar langer leeft met een nut van consumptie van 50.

Hieronder ziet u een figuur die deze situatie en het extra nut van consumptie door de levensverlenging door dit medicijn laat zien.

## B.3 Questions utility of consumption

Wij vragen u hierna aan te geven hoe hoog u denkt dat uw nut van consumptie zou zijn in verschillende gezondheidstoestanden.

### B.3.A Utility of consumption for quality of life 25

Stelt u zich voor dat u door ziekte het komende jaar leeft in een kwaliteit van leven van 25 op de schaal van 0 (dood) tot 100 (volledig gezond). Deze situatie is uitgebeeld in de figuur links hieronder.

Hoe hoog denkt u dat uw nut van consumptie zou zijn in dit jaar in deze gezondheidstoestand op de schaal van 0 (geen nut) tot 100 (hoogst haalbare nut))? Ga hierbij uit van uw huidige inkomen.

Geef op de schaal in de figuur rechts hieronder aan welk getal uw nut het beste weergeeft. Dit doet u door op het antwoordblokje te klikken en dit met uw muis ingedrukt naar boven of beneden te schuiven.

**<Open question, answer should be in range 0-100, answer should be visible in figure right below in a manner equal to figure left below (in that figure, answer would be 25)>**

### B.3.B Utility of consumption for quality of life 50

Stelt u zich voor dat u door ziekte het komende jaar leeft in een kwaliteit van leven van 50 op de schaal van 0 (dood) tot 100 (volledig gezond). Deze situatie is uitgebeeld in de figuur links hieronder.

Hoe hoog denkt u dat uw nut van consumptie is in dit jaar in deze gezondheidstoestand op de schaal van 0 (geen nut) tot 100 (hoogst haalbare nut))? Ga hierbij uit van uw huidige inkomen.

Geef op de schaal in de figuur rechts hieronder aan welk getal uw nut het beste weergeeft. Dit doet u door op het antwoordblokje te klikken en dit met uw muis ingedrukt naar boven of beneden te schuiven.

**<Open question, answer should be in range 0-100, answer should be visible in figure right below in a manner equal to figure left below (in that figure, answer would be 50)>**

### B.3.C Utility of consumption for quality of life 75

Stelt u zich voor dat u door ziekte het komende jaar leeft in een kwaliteit van leven van 75 op de schaal van 0 (dood) tot 100 (volledig gezond). Deze situatie is uitgebeeld in de figuur links hieronder.

Hoe hoog denkt u dat uw nut van consumptie is in dit jaar in deze gezondheidstoestand op de schaal van 0 (geen nut) tot 100 (hoogst haalbare nut))? Ga hierbij uit van uw huidige inkomen.

Geef op de schaal in de figuur rechts hieronder aan welk getal uw nut het beste weergeeft. Dit doet u door op het antwoordblokje te klikken en dit met uw muis ingedrukt naar boven of beneden te schuiven.

**<Open question, answer should be in range 0-100, answer should be visible in figure right below in a manner equal to figure left below (in that figure, answer would be 75)>**

### B.3.D Utility of consumption for quality of life 100

Stelt u zich voor dat u het komende jaar leeft in een kwaliteit van leven van 100 op de schaal van 0 (dood) tot 100 (volledig gezond). Deze situatie is uitgebeeld in de figuur links hieronder.

Hoe hoog denkt u dat uw nut van consumptie is in dit jaar in deze gezondheidstoestand op de schaal die loopt van 0 (geen nut) tot 100 (hoogst haalbare nut))? Ga hierbij uit van uw huidige inkomen.

Geef op de schaal in de figuur rechts hieronder aan welk getal uw nut het beste weergeeft. Dit doet u door op het antwoordblokje te klikken en dit met uw muis ingedrukt naar boven of beneden te schuiven.

**<Open question, answer should be in range 0-100, answer should be visible in figure right below in a manner equal to figure left below (in that figure, answer would be 100)>**

## B.4 WTP QoL: 25-50 [50% respondents answers questions for B.4 and B.6, other 50% answers questions for B.5 and B.7]

Er worden hierna steeds enkele scenario’s beschreven die vergelijkbaar zijn met de scenario’s in de voorbeelden hiervoor. Wij gebruiken hierin uw eerdere antwoorden over uw nut van consumptie in de verschillende gezondheidstoestanden. Wij stellen u vervolgens enkele vragen over wat u zou willen betalen voor het medicijn dat wordt beschreven in deze scenario’s.

### B.4.A WTP changes quality of life 25-50

Stelt u zich voor dat u door ziekte nog één jaar te leven heeft in een kwaliteit van leven van 25 op de schaal van 0 (dood) tot 100 (volledig gezond). Een medicijn kan ervoor zorgen dat uw kwaliteit van leven in dit jaar stijgt van 25 naar 50. Links hieronder ziet u een figuur die deze situatie en de stijging van de kwaliteit van leven door dit medicijn laat zien.

In de kwaliteit van leven van 25 heeft u een nut van consumptie van **<answer B.3.A>** op de schaal van 0 (geen nut) tot 100 (hoogst haalbare nut). In de kwaliteit van leven van 50 heeft u een nut van consumptie van **<answer B.3.B>**. Rechts hieronder ziet u een figuur die de stijging van het nut van consumptie door de stijging van de kwaliteit van leven door dit medicijn laat zien.

Voor deze stijging van kwaliteit van leven moet u het medicijn (zonder bijwerkingen) een jaar lang elke maand innemen. Uw kwaliteit van leven stijgt dan zeker van 25 naar 50 en uw nut van consumptie van **<answer B.3.A>** naar **<answer B.3.B>**. U moet het medicijn zelf betalen, uit uw eigen (gezins)inkomen. U betaalt in 12 maandelijkse termijnen. Uw inkomen is in de gehele periode gelijk aan uw huidige inkomen.

Wilt u de onderstaande bedragen afgaan, van laag naar hoog, en het hoogste bedrag kiezen dat u zeker wel **per maand** zou willen betalen voor dit medicijn? (bijvoorbeeld: als u zeker bent dat u €100 per maand zou willen betalen voor dit medicijn, maar niet zeker of u er €150 per maand voor over zou hebben, kiest u €100). Houd rekening met de invloed van de kwaliteit van leven op uw nut van consumptie.

| €0 | €10 | €15 | €25 | €50 | €75 | €100 | €125 | €150 | €200 | €250 | €300 | €500 | €750 | €1000 | €1500 | €2500 | … |
| --- | --- | --- | --- | --- | --- | --- | --- | --- | --- | --- | --- | --- | --- | --- | --- | --- | --- |
| O | O | O | O | O | O | O | O | O | O | O | O | O | O | O | O | O | O |

### B.4.B WTP changes quality of life 25-50

U heeft aangegeven dat u zeker 12 maanden [answer question B.4.A] per maand wilt betalen voor een medicijn dat zorgt dat uw kwaliteit van leven stijgt van 25 naar 50 op de schaal van 0 (dood) tot 100 (perfecte gezondheid) en als gevolg daarvan uw nut van consumptie van **<answer B.3.A>** naar **<answer B.3.B>** op de schaal van 0 (geen nut) tot 100 (hoogst haalbare nut).

Wilt u de onderstaande bedragen afgaan, van laag naar hoog, en het laagste bedrag kiezen dat u zeker niet **per maand** zou willen betalen voor dit medicijn? (bijvoorbeeld: als u zeker bent dat u geen €1.000 per maand zou willen betalen voor dit medicijn, maar er misschien wel €750 per maand voor over zou hebben, kiest u €1.000). Houd rekening met de invloed van de kwaliteit van leven op uw nut van consumptie.

| €0 | €10 | €15 | €25 | €50 | €75 | €100 | €125 | €150 | €200 | €250 | €300 | €500 | €750 | €1000 | €1500 | €2500 | … |
| --- | --- | --- | --- | --- | --- | --- | --- | --- | --- | --- | --- | --- | --- | --- | --- | --- | --- |
| O | O | O | O | O | O | O | O | O | O | O | O | O | O | O | O | O | O |

### B.4.C WTP changes quality of life 25-50

U heeft aangegeven dat u zeker 12 maanden [answer question B.4.A] per maand, maar zeker geen [answer question B.4.B] per maand wilt betalen voor een medicijn dat zorgt dat uw kwaliteit van leven stijgt van 25 naar 50 op de schaal van 0 (dood) tot 100 (perfecte gezondheid) en als gevolg daarvan uw nut van consumptie van **<answer B.3.A>** naar **<answer B.3.B>** op de schaal van 0 (geen nut) tot 100 (hoogst haalbare nut).

Wilt u het bedrag aangeven (tussen [answer question B.4.A] en [answer question B.4.B]) dat het maximale wat u bereid bent om te betalen voor dit medicijn het best benadert? Houd rekening met de invloed van de kwaliteit van leven op uw nut van consumptie.

**< open question, answer should be a number within the range [answer question B.4.A] - [answer question B.4.B >**

### B.4.D WTP changes quality of life 25-50 [only if B.4.C =0]

U heeft aangegeven dat u niet bereid bent meer dan €0 te betalen voor een medicijn dat zorgt dat uw kwaliteit van leven stijgt van 25 naar 50 op de schaal van 0 (dood) tot 100 (perfecte gezondheid) en als gevolg daarvan uw nut van consumptie van **<answer B.3.A>** naar **<answer B.3.B>** op de schaal van 0 (geen nut) tot 100 (hoogst haalbare nut)). Wat is uw belangrijkste reden hiervoor?

- Ik kan niet meer dan €0 betalen
- Medicijnen zijn niet meer dan €0 waard voor mij
- Ik vind de medicijnen meer dan €0 waard, maar geef mijn geld liever uit aan iets anders
- Ik vind dat deze kosten vanuit de basis zorgverzekering zouden moeten worden vergoed
- Ik gebruik (liever) geen medicijnen
- De waarde van zorg en gezondheid is voor mij niet in geld uit te drukken
- Anders, namelijk **<open question >**

## B.5 WTP QoL: 75-100 [50% respondents answers questions for B.4 and B.6, other 50% answers questions for B.5 and B.7]

### B.5.A WTP changes quality of life 75-100

Stelt u zich voor dat u door ziekte nog één jaar te leven heeft in een kwaliteit van leven van 75 op de schaal van 0 (dood) tot 100 (volledig gezond). Een medicijn kan ervoor zorgen dat uw kwaliteit van leven in dit jaar stijgt van 75 naar 100. Links hieronder ziet u een figuur die deze situatie en de stijging van de kwaliteit van leven door dit medicijn laat zien.

In de kwaliteit van leven van 75 heeft u een nut van consumptie van **<answer B.3.C>** op de schaal van 0 (geen nut) tot 100 (hoogst haalbare nut). In de kwaliteit van leven van 100 heeft u een nut van consumptie van **<answer B.3.D>**. Rechts hieronder ziet u een figuur die de stijging van het nut van consumptie door de stijging van de kwaliteit van leven door dit medicijn laat zien.

Voor deze stijging van kwaliteit van leven moet u het medicijn (zonder bijwerkingen) een jaar lang elke maand innemen. Uw kwaliteit van leven stijgt dan zeker van 75 naar 100 en uw nut van consumptie van **<answer B.3.C>** naar **<answer B.3.D>**. U moet het medicijn zelf betalen, uit uw eigen (gezins)inkomen. U betaalt in 12 maandelijkse termijnen. Uw inkomen is in de gehele periode gelijk aan uw huidige inkomen.

Wilt u de onderstaande bedragen afgaan, van laag naar hoog, en het hoogste bedrag kiezen dat u zeker wel **per maand** zou willen betalen voor dit medicijn? (bijvoorbeeld: als u zeker bent dat u €100 per maand zou willen betalen voor dit medicijn, maar niet zeker of u er €150 per maand voor over zou hebben, kiest u €100). Houd rekening met de invloed van de kwaliteit van leven op uw nut van consumptie.

| €0 | €10 | €15 | €25 | €50 | €75 | €100 | €125 | €150 | €200 | €250 | €300 | €500 | €750 | €1000 | €1500 | €2500 | … |
| --- | --- | --- | --- | --- | --- | --- | --- | --- | --- | --- | --- | --- | --- | --- | --- | --- | --- |
| O | O | O | O | O | O | O | O | O | O | O | O | O | O | O | O | O | O |

### B.5.B WTP changes quality of life 75-100

U heeft aangegeven dat u zeker 12 maanden [answer question B.5.A] per maand wilt betalen voor een medicijn dat zorgt dat uw kwaliteit van leven stijgt van 75 naar 100 op de schaal van 0 (dood) tot 100 (perfecte gezondheid) en uw nut van consumptie van **<answer B.3.C>** naar **<answer B.3.D>** op de schaal van 0 (geen nut) tot 100 (hoogst haalbare nut).

Wilt u de onderstaande bedragen afgaan, van laag naar hoog, en het laagste bedrag kiezen dat u zeker niet **per maand** zou willen betalen voor dit medicijn? (bijvoorbeeld: als u zeker bent dat u geen €1.000 per maand zou willen betalen voor dit medicijn, maar er misschien wel €750 per maand voor over zou hebben, kiest u €1.000). Houd rekening met de invloed van de kwaliteit van leven op uw nut van consumptie.

| €0 | €10 | €15 | €25 | €50 | €75 | €100 | €125 | €150 | €200 | €250 | €300 | €500 | €750 | €1000 | €1500 | €2500 | … |
| --- | --- | --- | --- | --- | --- | --- | --- | --- | --- | --- | --- | --- | --- | --- | --- | --- | --- |
| O | O | O | O | O | O | O | O | O | O | O | O | O | O | O | O | O | O |

### B.5.C WTP changes quality of life 75-100

U heeft aangegeven dat u zeker 12 maanden [answer question B.5.A] per maand, maar zeker geen [answer question B.5.B] per maand wilt betalen voor een medicijn dat zorgt dat uw kwaliteit van leven stijgt van 75 naar 100 op de schaal van 0 (dood) tot 100 (perfecte gezondheid) en uw nut van consumptie van **<answer B.3.C>** naar **<answer B.3.D>** op de schaal van 0 (geen nut) tot 100 (hoogst haalbare nut).

Wilt u het bedrag aangeven (tussen [answer question B.5.A] en [answer question B.5.B]) dat het maximale wat u bereid bent om te betalen voor dit medicijn het best benadert? Houd rekening met de invloed van de kwaliteit van leven op uw nut van consumptie.

**< open question, answer should be a number within the range [answer question B.5.A] - [answer question B.5.B >**

### B.5.D WTP changes quality of life 75-100 [only if B.5.C =0]

U heeft aangegeven dat u niet bereid bent meer dan €0 te betalen voor een medicijn dat zorgt dat uw kwaliteit van leven stijgt van 75 naar 100 op de schaal van 0 (dood) tot 100 (perfecte gezondheid) en als gevolg daarvan uw nut van consumptie van **<answer B.3.A>** naar **<answer B.3.B>** op de schaal van 0 (geen nut) tot 100 (hoogst haalbare nut)). Wat is uw belangrijkste reden hiervoor?

- Ik kan niet meer dan €0 betalen
- Medicijnen zijn niet meer dan €0 waard voor mij
- Ik vind de medicijnen meer dan €0 waard, maar geef mijn geld liever uit aan iets anders
- Ik vind dat deze kosten vanuit de basis zorgverzekering zouden moeten worden vergoed
- Ik gebruik (liever) geen medicijnen
- De waarde van zorg en gezondheid is voor mij niet in geld uit te drukken
- Anders, namelijk **<open question >**

## B.6 WTP LL: +25, 1 year [50% respondents answers questions for B.4 and B.6, other 50% answers questions for B.5 and B.7]

### B.6.A WTP changes length of life +25, 1 year

Stelt u zich voor dat u door ziekte nog één jaar te leven heeft in een kwaliteit van leven van 25 op de schaal van 0 (dood) tot 100 (volledig gezond). Een medicijn kan ervoor zorgen dat u één jaar langer leeft in kwaliteit van leven van 25. Links hieronder ziet u een figuur die deze situatie en de levensverlenging door dit medicijn laat zien.

In de kwaliteit van leven van 25 heeft u een nut van consumptie van **<answer B.3.A>** op de schaal van 0 (geen nut) tot 100 (hoogst haalbare nut). Rechts hieronder ziet u een figuur die deze situatie en het extra nut van consumptie door de levensverlenging door dit medicijn laat zien.

Voor deze levensverlenging moet u het medicijn (zonder bijwerkingen) een jaar lang elke maand innemen. U leeft dan zeker één jaar langer in een kwaliteit van leven van 25 met een nut van consumptie van **<answer B.3.A>**. U moet het medicijn zelf betalen, uit uw eigen (gezins)inkomen. De betaling vindt plaats in 12 maandelijkse termijnen (u betaalt dus niet voor dit medicijn in de periode dat u langer leeft). Uw inkomen is in de gehele periode gelijk aan uw huidige inkomen.

Wilt u de onderstaande bedragen afgaan, van laag naar hoog, en het hoogste bedrag kiezen dat u zeker wel **per maand** zou willen betalen voor dit medicijn? (bijvoorbeeld: als u zeker bent dat u €100 per maand zou willen betalen voor dit medicijn, maar niet zeker of u er €150 per maand voor over zou hebben, kiest u €100). Houd rekening met de invloed van de kwaliteit van leven op uw nut van consumptie.

| €0 | €10 | €15 | €25 | €50 | €75 | €100 | €125 | €150 | €200 | €250 | €300 | €500 | €750 | €1000 | €1500 | €2500 | … |
| --- | --- | --- | --- | --- | --- | --- | --- | --- | --- | --- | --- | --- | --- | --- | --- | --- | --- |
| O | O | O | O | O | O | O | O | O | O | O | O | O | O | O | O | O | O |

### B.6.B WTP changes length of life +25, 1 year

U heeft aangegeven dat u zeker 12 maanden [answer question B.6.A] per maand wilt betalen voor een medicijn dat zorgt dat u één jaar langer leeft in kwaliteit van leven van 25 op de schaal van 0 (dood) tot 100 (perfecte gezondheid) met een nut van consumptie van **<answer B.3.A>** op de schaal van 0 (geen nut) tot 100 (hoogst haalbare nut).

Wilt u de onderstaande bedragen afgaan, van laag naar hoog, en het laagste bedrag kiezen dat u zeker niet **per maand** zou willen betalen voor dit medicijn? (bijvoorbeeld: als u zeker bent dat u geen €1.000 per maand zou willen betalen voor dit medicijn, maar er misschien wel €750 per maand voor over zou hebben, kiest u €1.000). Houd rekening met de invloed van de kwaliteit van leven op uw nut van consumptie.

| €0 | €10 | €15 | €25 | €50 | €75 | €100 | €125 | €150 | €200 | €250 | €300 | €500 | €750 | €1000 | €1500 | €2500 | … |
| --- | --- | --- | --- | --- | --- | --- | --- | --- | --- | --- | --- | --- | --- | --- | --- | --- | --- |
| O | O | O | O | O | O | O | O | O | O | O | O | O | O | O | O | O | O |

### B.6.C WTP changes length of life +25, 1 year

U heeft aangegeven dat u zeker 12 maanden [answer question B.6.A] per maand, maar zeker geen [answer question B.6.B] per maand wilt betalen voor een medicijn dat zorgt dat u één jaar langer leeft in kwaliteit van leven van 25 op de schaal van 0 (dood) tot 100 (perfecte gezondheid) met een nut van consumptie van **<answer B.3.A>** op de schaal van 0 (geen nut) tot 100 (hoogst haalbare nut).

Wilt u het bedrag aangeven (tussen [answer question B.6.A] en [answer question B.6.B]) dat het maximale wat u bereid bent om te betalen voor dit medicijn het best benadert? Houd rekening met de invloed van de kwaliteit van leven op uw nut van consumptie.

**< open question, answer should be a number within the range [answer question B.6.A] - [answer question B.6.B >**

### B.6.D WTP changes length of life +25, 1 year [only if B.6.C =0]

U heeft aangegeven dat u niet bereid bent meer dan €0 te betalen voor een medicijn dat ervoor zorgt dat u één jaar langer leeft in kwaliteit van leven van 25 op de schaal van 0 (dood) tot 100 (perfecte gezondheid) met een nut van consumptie van **<answer B.3.A>** op de schaal van 0 (geen nut) tot 100 (hoogst haalbare nut)). Wat is uw belangrijkste reden hiervoor?

- Ik kan niet meer dan €0 betalen
- Medicijnen zijn niet meer dan €0 waard voor mij
- Ik vind de medicijnen meer dan €0 waard, maar geef mijn geld liever uit aan iets anders
- Ik vind dat deze kosten vanuit de basis zorgverzekering zouden moeten worden vergoed
- Ik gebruik (liever) geen medicijnen
- De waarde van zorg en gezondheid is voor mij niet in geld uit te drukken
- Anders, namelijk **<open question >**

## B.7 WTP LL: +75, 4 months [50% respondents answers questions for B.4 and B.6, other 50% answers questions for B.5 and B.7]

### B.7.A WTP changes length of life +75, 4 months

Stelt u zich voor dat u door ziekte nog één jaar te leven heeft in een kwaliteit van leven van 75 op de schaal van 0 (dood) tot 100 (volledig gezond). Een medicijn kan ervoor zorgen dat u vier maanden langer leeft in kwaliteit van leven van 75. Hieronder ziet u een figuur die deze situatie en de levensverlenging door dit medicijn laat zien.

In de kwaliteit van leven van 75 heeft u een nut van consumptie van **<answer B.3.C>** op de schaal van 0 (geen nut) tot 100 (hoogst haalbare nut). Rechts hieronder ziet u een figuur die deze situatie en het extra nut van consumptie door de levensverlenging door dit medicijn laat zien.

Voor deze levensverlenging moet u het medicijn (zonder bijwerkingen) een jaar lang elke maand innemen. U leeft dan zeker vier maanden langer in een kwaliteit van leven van 75 met een nut van consumptie van **<answer B.3.C>**. U moet het medicijn zelf betalen, uit uw eigen (gezins)inkomen. De betaling vindt plaats in 12 maandelijkse termijnen (u betaalt dus niet voor dit medicijn in de periode dat u langer leeft). Uw inkomen is in de gehele periode gelijk aan uw huidige inkomen.

Wilt u de onderstaande bedragen afgaan, van laag naar hoog, en het hoogste bedrag kiezen dat u zeker wel **per maand** zou willen betalen voor dit medicijn? (bijvoorbeeld: als u zeker bent dat u €100 per maand zou willen betalen voor dit medicijn, maar niet zeker of u er €150 per maand voor over zou hebben, kiest u €100). Houd rekening met de invloed van de kwaliteit van leven op uw nut van consumptie.

| €0 | €10 | €15 | €25 | €50 | €75 | €100 | €125 | €150 | €200 | €250 | €300 | €500 | €750 | €1000 | €1500 | €2500 | … |
| --- | --- | --- | --- | --- | --- | --- | --- | --- | --- | --- | --- | --- | --- | --- | --- | --- | --- |
| O | O | O | O | O | O | O | O | O | O | O | O | O | O | O | O | O | O |

### B.7.B WTP changes length of life +75, 4 months

U heeft aangegeven dat u zeker 12 maanden [answer question B.7.A] per maand wilt betalen voor een medicijn dat zorgt dat u vier maanden langer leeft in kwaliteit van leven van 75 op de schaal van 0 (dood) tot 100 (perfecte gezondheid) met een nut van consumptie van **<answer B.3.C>** op de schaal van 0 (geen nut) tot 100 (hoogst haalbare nut)).

Wilt u de onderstaande bedragen afgaan, van laag naar hoog, en het laagste bedrag kiezen dat u zeker niet **per maand** zou willen betalen voor dit medicijn? (bijvoorbeeld: als u zeker bent dat u geen €1.000 per maand zou willen betalen voor dit medicijn, maar er misschien wel €750 per maand voor over zou hebben, kiest u €1.000). Houd rekening met de invloed van de kwaliteit van leven op uw nut van consumptie.

| €0 | €10 | €15 | €25 | €50 | €75 | €100 | €125 | €150 | €200 | €250 | €300 | €500 | €750 | €1000 | €1500 | €2500 | … |
| --- | --- | --- | --- | --- | --- | --- | --- | --- | --- | --- | --- | --- | --- | --- | --- | --- | --- |
| O | O | O | O | O | O | O | O | O | O | O | O | O | O | O | O | O | O |

### B.7.C WTP changes length of life +75, 4 months

U heeft aangegeven dat u zeker 12 maanden [answer question B.7.A] per maand, maar zeker geen [answer question B.7.B] per maand wilt betalen voor een medicijn dat zorgt dat u vier maanden langer leeft in kwaliteit van leven van 75 op de schaal van 0 (dood) tot 100 (perfecte gezondheid) met een nut van consumptie van **<answer B.3.C>** op de schaal van 0 (geen nut) tot 100 (hoogst haalbare nut).

Wilt u het bedrag aangeven (tussen [answer question B.7.A] en [answer question B.7.B]) dat het maximale wat u bereid bent om te betalen voor dit medicijn het best benadert? Houd rekening met de invloed van de kwaliteit van leven op uw nut van consumptie.

**< open question, answer should be a number within the range [answer question B.7.A] - [answer question B.7.B >**

### B.7.D WTP changes length of life +75, 4 months [only if B.7.C =0]

U heeft aangegeven dat u niet bereid bent meer dan €0 te betalen voor een medicijn dat ervoor zorgt dat u vier maanden langer leeft in kwaliteit van leven van 75 op de schaal van 0 (dood) tot 100 (perfecte gezondheid) met een nut van consumptie van **<answer B.3.C>** op de schaal van 0 (geen nut) tot 100 (hoogst haalbare nut). Wat is uw belangrijkste reden hiervoor?

- Ik kan niet meer dan €0 betalen
- Medicijnen zijn niet meer dan €0 waard voor mij
- Ik vind de medicijnen meer dan €0 waard, maar geef mijn geld liever uit aan iets anders
- Ik vind dat deze kosten vanuit de basis zorgverzekering zouden moeten worden vergoed
- Ik gebruik (liever) geen medicijnen
- De waarde van zorg en gezondheid is voor mij niet in geld uit te drukken
- Anders, namelijk **<open question >**

# C: Part 2 - Follow up questions WTP

Dit was het eerste deel van de vragenlijst. In het tweede deel van de vragenlijst stellen wij u enkele vragen over de zaken waar u rekening mee heeft gehouden in het eerste deel van de vragenlijst.

## C.1 Follow-up WTP [multiple answers possible]

U heeft in het eerste deel van de vragenlijst aangegeven wat u zou willen betalen voor verschillende medicijnen. Met welke zaken hield u hierbij rekening? U kunt meerdere antwoorden kiezen.

- Mijn productiviteit in de verschillende situaties (hoe goed ik in de verschillende situaties mijn werk kan doen)
- De tijd beschikbaar voor vrije tijd in de verschillende situaties
- Mijn bestedingspatroon in de verschillende situaties (de manier waarop ik in de verschillende situaties mijn geld besteed)
- Geen van bovenstaande opties

### C.1.B Follow-up WTP - productivity

#### **C.1.B.1 Follow-up WTP – productivity 1** [only if option 2 in C.1 is chosen]

U gaf aan dat u ook dacht aan uw **productiviteit** (hoe goed u uw werk zou kunnen doen) bij het beantwoorden van de vragen. Wat waren uw verwachtingen over de invloed van veranderingen in uw kwaliteit van leven op uw productiviteit? Kies het antwoord dat het beste past.

- Ik verwachtte dat mijn productiviteit *niet zou veranderen*
- Ik verwachtte dat een slechtere gezondheid mijn productiviteit *zou verlagen*
- Ik verwachtte dat een slechtere gezondheid mijn productiviteit *zou verhogen*
- Anders, namelijk <open question>

#### **C.1.B.2 Follow-up WTP – productivity 2** [only if option 2 in C.1 is chosen]

Wat waren uw verwachtingen over uw **productiviteit** in de verschillende situaties waar u langer zou leven (het gaat hier dus over uw productiviteit in de periode dat u extra zou leven)? Kies het antwoord dat het beste past.

- Ik verwachtte dat mijn productiviteit *gelijk* zou zijn aan mijn productiviteit in het jaar daarvoor
- Ik verwachtte dat mijn productiviteit *lager* zou zijn dan mijn productiviteit in het jaar daarvoor
- Ik verwachtte dat mijn productiviteit *hoger* zou zijn dan mijn productiviteit in het jaar daarvoor
- Anders, namelijk <open question>

#### **C.1.B.3 Follow-up WTP – productivity 3** [only if option 2 in C.1 is chosen]

In hoeverre heeft u uw **productiviteit** in de verschillende situaties laten meewegen in uw antwoorden? Kies het antwoord dat het beste past.

- Productiviteit heeft mijn antwoorden *niet of nauwelijks* beïnvloed
- Productiviteit heeft mijn antwoorden *een beetje* beïnvloed
- Productiviteit heeft mijn antwoorden *sterk* beïnvloed

#### **C.1.B.4 Follow-up WTP – productivity 4** [only if option 2 in C.1 is NOT chosen]

U gaf aan dat u niet dacht aan uw **productiviteit** bij het beantwoorden van de vragen. Wat is hiervoor uw reden? Kies het antwoord dat het beste past.

- Dit werd niet specifiek gevraagd
- Er was geen informatie beschikbaar over productiviteit
- Ik heb hier niet over nagedacht
- Anders, namelijk **<open question>**

### C.1.C Follow-up WTP - leisure

#### **C.1.C.1 Follow-up WTP – leisure 1** [only if option 3 in C.1 is chosen]

U gaf aan dat u ook dacht aan de **tijd beschikbaar voor vrije tijd** bij het beantwoorden van de vragen. Wat waren uw verwachtingen over de invloed van veranderingen in uw kwaliteit van leven op uw tijd beschikbaar voor vrije tijd? Kies het antwoord dat het beste past.

- Ik verwachtte dat de tijd beschikbaar voor vrije tijd *niet zou veranderen*
- Ik verwachtte dat een slechtere gezondheid zou zorgen voor *minder* tijd beschikbaar voor vrije tijd
- Ik verwachtte dat een slechtere gezondheid zou zorgen voor *meer* tijd beschikbaar voor vrije tijd
- Anders, namelijk <open question>

#### **C.1.C.2 Follow-up WTP – leisure 2** [only if option 3 in C.1 is chosen]

Wat waren uw verwachtingen over de **tijd beschikbaar voor vrije tijd** in de verschillende situaties waar u langer zou leven (het gaat hier dus over de tijd beschikbaar voor vrije tijd in de periode dat u extra zou leven)? Kies het antwoord dat het beste past.

- Ik verwachtte dat ik *evenveel* tijd beschikbaar zou hebben voor vrije tijd als in het jaar daarvoor
- Ik verwachtte dat ik *minder* tijd beschikbaar zou hebben voor vrije tijd dan in het jaar daarvoor
- Ik verwachtte dat ik *meer* tijd beschikbaar zou hebben voor vrije tijd dan in het jaar daarvoor
- Anders, namelijk <open question>

#### **C.1.C.3 Follow-up WTP – leisure 3** [only if option 3 in C.1 is chosen]

In hoeverre heeft u de **tijd beschikbaar voor vrije tijd** in de verschillende situaties laten meewegen in uw antwoorden? Kies het antwoord dat het beste past.

- De tijd beschikbaar voor vrije tijd heeft mijn antwoorden *niet of nauwelijks* beïnvloed
- De tijd beschikbaar voor vrije tijd heeft mijn antwoorden *een beetje* beïnvloed
- De tijd beschikbaar voor vrije tijd heeft mijn antwoorden *sterk* beïnvloed

#### **C.1.C.4 Follow-up WTP – leisure 4** [only if option 3 in C.1 is NOT chosen]

U gaf aan dat u niet dacht aan de **tijd beschikbaar voor vrije tijd** bij het beantwoorden van de vragen. Wat is hiervoor uw reden? Kies het antwoord dat het beste past.

- Dit werd niet specifiek gevraagd
- Er was geen informatie beschikbaar over de tijd beschikbaar voor vrije tijd
- Ik heb hier niet over nagedacht
- Anders, namelijk **<open question>**

### **C.1.D Follow-up WTP - consumption**

#### **C.1.D.1 Follow-up WTP – consumption 1** [only if option 4 in C.1 is chosen]

U gaf aan dat u ook dacht aan uw **bestedingspatroon** (de manier waarop u uw geld zou besteden) bij het beantwoorden van de vragen. Wat waren uw verwachtingen over de invloed van veranderingen in uw kwaliteit van leven op uw bestedingspatroon? Kies het antwoord dat het beste past.

- Ik verwachtte dat mijn bestedingspatroon *niet zou veranderen*
- Ik verwachtte dat ik in een slechtere gezondheid in totaal *minder* zou besteden
- Ik verwachtte dat ik in een slechtere gezondheid in totaal *meer* zou besteden
- Ik verwachtte dat ik in totaal *evenveel* zou besteden in een slechtere gezondheid, *maar dat ik mijn geld wel aan andere dingen zou uitgeven*
- Anders, namelijk **<open question>**

#### **C.1.D.2 Follow-up WTP – consumption 2** [only if option 4 in C.1 is chosen AND option 2, 3, 4, or 5 in C.1.D.1]

Op welke van de volgende categorieën bestedingen dacht u dat veranderingen in uw kwaliteit van leven invloed zouden hebben? U kunt meerdere antwoorden kiezen.

- Voeding
- Dranken en rookwaren
- Kleding en schoeisel
- Wonen
- Woninginrichting en huisraad
- Gezondheidszorg
- Vervoer en communicatie
- Recreatie en ontwikkeling
- Overig

#### **C.1.D.3 Follow-up WTP – consumption 3** [only if option 4 in C.1 is chosen AND option 2, 3, 4, or 5 in C.1.D.1]

U gaf aan dat de u dacht dat de veranderingen in uw kwaliteit van leven invloed zouden hebben op de categorie(ën) hieronder. Zou u meer of minder uitgeven aan deze categorie(ën)?

**Meer** **Minder**

- Voeding □ □
- Dranken en rookwaren □ □
- Kleding en schoeisel □ □
- Wonen □ □
- Woninginrichting en huisraad □ □
- Gezondheidszorg □ □
- Vervoer en communicatie □ □
- Recreatie en ontwikkeling □ □
- Overig □ □

#### **C.1.D.4 Follow-up WTP – consumption 4** [only if option 4 in C.1 is chosen]

Wat waren uw verwachtingen over uw **bestedingspatroon** in de verschillende situaties waar u langer zou leven (het gaat hier dus over uw bestedingspatroon in de periode dat u extra zou leven)? Kies het antwoord dat het beste past.

- Ik verwachtte dat mijn bestedingspatroon *gelijk* zou zijn aan dat in het jaar daarvoor
- Ik verwachtte dat ik in totaal *minder* zou besteden dan in het jaar daarvoor
- Ik verwachtte dat ik in totaal *meer* zou besteden dan in het jaar daarvoor
- Ik verwachtte dat ik in totaal *evenveel* zou besteden als in het jaar daarvoor, *maar dat ik mijn geld wel aan andere dingen zou uitgeven*
- Anders, namelijk <open question>

#### **C.1.D.5 Follow-up WTP – consumption 5** [only if option 4 in C.1 is chosen AND option 2, 3, 4, or 5 in C.1.D.4]

Op welke van de volgende categorieën bestedingen dacht u dat de verschillende situaties (waar u langer zou leven) invloed hadden? U kunt meerdere antwoorden kiezen.

- Voeding
- Dranken en rookwaren
- Kleding en schoeisel
- Wonen
- Woninginrichting en huisraad
- Gezondheidszorg
- Vervoer en communicatie
- Recreatie en ontwikkeling
- Overig

#### **C.1.D.6 Follow-up WTP – consumption 6** [only if option 4 in C.1 is chosen AND option 2, 3, 4, or 5 in C.1.D.4]

U gaf aan dat de situaties (waar u langer zou leven) uw bestedingen in de categorie(ën) hieronder zou beïnvloeden. Zou u meer of minder uitgeven aan deze categorie(ën)?

**Meer** **Minder**

- Voeding □ □
- Dranken en rookwaren □ □
- Kleding en schoeisel □ □
- Wonen □ □
- Woninginrichting en huisraad □ □
- Gezondheidszorg □ □
- Vervoer en communicatie □ □
- Recreatie en ontwikkeling □ □
- Overig □ □

#### **C.1.D.7 Follow-up WTP – consumption 7** [only if option 4 in C.1 is chosen]

In hoeverre heeft u uw **bestedingspatroon** in de verschillende situaties laten meewegen in uw antwoorden? Kies het antwoord dat het beste past.

- Mijn bestedingspatroon heeft mijn antwoorden *niet of nauwelijks* beïnvloed
- Mijn bestedingspatroon heeft mijn antwoorden *een beetje* beïnvloed
- Mijn bestedingspatroon heeft mijn antwoorden *sterk* beïnvloed

#### **C.1.D.8 Follow-up WTP – consumption 8** [only if option 4 in C.1 is NOT chosen]

U gaf aan dat u niet dacht aan uw **bestedingspatroon** bij het beantwoorden van de vragen. Wat is hiervoor uw reden? Kies het antwoord dat het beste past.

- Dit werd niet specifiek gevraagd
- Er was geen informatie beschikbaar over bestedingspatronen
- Ik heb hier niet over nagedacht
- Anders, namelijk **<open question>**

## C.2 Questions income for changing health

### C.2.A Expectations income for changing health QoL

Wij hebben u hiervoor steeds gevraagd om uit te gaan van uw huidige inkomen. Hoe denkt u dat uw inkomen in het komende jaar zou veranderen als uw kwaliteit van leven zou veranderen? Kies het antwoord dat het beste past.

- Ik verwacht dat mijn inkomen *niet zou veranderen*
- Ik verwacht dat een slechtere gezondheid mijn inkomen *zou verlagen*
- Ik verwacht dat een slechtere gezondheid mijn inkomen *zou verhogen*
- Anders, namelijk <open question>

### C.2.B. Expectations income for changing health Length

Wij hebben u ook gevraagd om uit te gaan van uw huidige inkomen wanneer wij u vroegen u voor te stellen dat u door een medicijn langer zou leven. Wat denkt u dat uw inkomen zou zijn in de periode dat u langer zou leven? Kies het antwoord dat het beste past.

- Ik verwacht dat mijn inkomen *gelijk* zou zijn aan mijn inkomen in het jaar daarvoor
- Ik verwacht dat mijn inkomen *lager* zou zijn dan mijn inkomen in het jaar daarvoor
- Ik verwacht dat mijn inkomen *hoger* zou zijn dan mijn inkomen in het jaar daarvoor
- Anders, namelijk <open question>

# D: Part 3 - Respondent information

Dit was het tweede deel van de vragenlijst. In het derde en laatste deel stellen wij u enkele vragen over uzelf.

## D.1 Age

Wat is uw leeftijd?

**<open question, answer must be a number>**

## D.2 Gender

Wat is uw geslacht?

- Man
- Vrouw

## D.3 Highest education

Wat is uw hoogst afgeronde opleiding?

- Geen opleiding
- Lagere school/ Basisonderwijs
- Lager of voorbereidend beroepsonderwijs: LBO, VBO, LTS. LHNO, VMBO
- Middelbaar algemeen voortgezet onderwijs: MAVO, VMBO-t, MBO-kort
- Middelbaar beroepsonderwijs: MBO, MTS, MEAO
- Hoger algemeen voortgezet onderwijs: HAVO, VWO, Gymnasium
- Hoger beroepsonderwijs: HBO, HEAO, HTS
- Wetenschappelijk onderwijs: Universiteit
- Anders, namelijk **<open question>**

## D.4 Marital status

Wat is uw huwelijkse staat?

- Alleenstaand, nooit getrouwd
- Samenwonend met partner
- Getrouwd, geregistreerd partnerschap
- Gescheiden
- Weduwe, weduwnaar

## D.5 Household size

Uit hoeveel personen bestaat uw huishouden? Reken uzelf mee.

**<open question, answer must be a number, minimum 1>**

## D.6 Children

Heeft u kinderen?

- Ja, ik heb **<open question, answer must be a number, minimum of 1>** kind(eren)
- Nee

## D.7 Children at home [only if D.6 = ‘Ja…’]

Hoeveel kinderen onder de 18 jaar wonen (deeltijd) bij u thuis?

❑ **<open question, answer must be a number>** kind(eren)

## D.8 Employment

Kies hieronder wat voor u tijdens een werkweek het meest van toepassing is. Waaraan besteedt u het meeste van uw tijd tijdens een werkweek?

- Ik ben in loondienst
- Ik ben zelfstandig ondernemer
- Ik ben werkloos, werkzoekend
- Ik ben met de VUT, (pre)pensioen of rentenierend
- Ik ben studerend, schoolgaand
- Ik ben huisman, huisvrouw
- Ik doe vrijwilligerswerk
- Anders, namelijk **<open question >**

## D.9 Net household income

Wat is het netto-inkomen per maand van uw huishouden?

**<open question, answer must be a number>**

## D.10 Income sufficient

Als u denkt aan het totale inkomen per maand van uw huishouden, hoe goed kan uw huishouden rondkomen?

- Met veel moeite
- Met enige moeite
- Vrij gemakkelijk
- Gemakkelijk

## D.11 Religion 1

Wat is uw geloofsovertuiging?

- Ik ben niet gelovig/atheïst/agnost
- Protestantschristelijk
- Katholiek christelijk
- Islamitisch
- Boeddhistisch
- Hindoeïstisch
- Anders, namelijk **<open question>**

## D.12 Religion 2

Hoe belangrijk is geloof voor u in uw dagelijkse leven?

- Volledig onbelangrijk
- Onbelangrijk
- Neutraal
- Belangrijk
- Zeer belangrijk

# E: End questionnaire

Dit is het einde van de vragenlijst. Bedankt voor uw deelname!
